# Enhancing network stability in VANETs using nature inspired algorithm for intelligent transportation system

**DOI:** 10.1371/journal.pone.0296331

**Published:** 2024-01-11

**Authors:** Sandeep Yerrathi, Venugopal Pakala

**Affiliations:** School of Electronics Engineering, Vellore Institute of Technology, Vellore, Tamil Nadu, India; Sri Eshwar College of Engineering, INDIA

## Abstract

The Internet of Vehicles (IoV) is one of the developing paradigms that integrates the automotive industry with the Internet of Things (IoT). The evolution of traditional Vehicular Ad-hoc Networks (VANETs), which are a layered framework for Intelligent Transportation Systems (ITS), is employed to provide Quality of Service (QoS) to end users in hazardous situations. VANETs can set up ad-hoc networks and share information among themselves using Peer-to-Peer (P2P) communication. Dynamic properties in VANETs such as dynamic topology, node mobility, sparse vehicle distribution, and bandwidth constraints can have an impact on scalability, routing, and security. This can result in frequent link failures, instability, reliability, and QOS concerns, as well as the inherent complexity of NP-hard problems. Researchers have proposed several techniques to achieve stability; the most prominent one is clustering, which relies on mobility metrics. However, existing clustering techniques generate overwhelming clusters, resulting in greater resource consumption, communication overhead, and hop count, which may lead to increased latency. Therefore, the primary objective is to achieve stability by increasing cluster lifetime, which is accomplished by generating optimal clusters. A nature-inspired meta-heuristic algorithm titled African Vulture Optimization Based Clustering Algorithm (AVOCA) is implemented to achieve it. The proposed algorithm can achieve load optimization with efficient resource utilization by mitigating hidden node challenges and ensuring communication proficiency. By maintaining an equilibrium state between the exploration and exploitation phases, AVOCA avoids local optima. The paper explores a taxonomy of the techniques used in Cluster Head (CH) selection, coordination, and maintenance to achieve stability with lower communication costs. We evaluated the effectiveness of AVOCA using various network grid sizes, transmission ranges, and network nodes. The results show that AVOCA generates 40% less clusters when compared to the Clustering Algorithm Based on Moth-Flame Optimization for VANETs (CAMONET). AVOCA generates 45% less clusters when compared to Self-Adaptive Multi-Kernel Clustering for Urban VANETs (SAMNET), AVOCA generates 43% less clusters when compared to Intelligent Whale Optimization Algorithm (i-WOA) and AVOCA generates 38% less clusters when compared to Harris Hawks Optimization (HHO). The results show that AVOCA outperforms state-of-the-art algorithms in generating optimal clusters.

## 1. Introduction

The number of vehicles on every road in sustainable cities keeps growing day by day, and as a result, accidents and traffic congestion are rising rapidly [1]. This has led to the emergence of significant industrial and scientific projects by researchers and engineers worldwide to accomplish augmentation in ITS [2]. In the current era of IoT, every vehicle that has access to the Internet can share data within the network is referred to as an IOV, which enables smart city functionalities [3]. The IOV heterogeneous framework has enormous potential, and capability, and is now peering over the horizon to supervise and steer vehicles for an abundance of applications [4]. The evolution of multiple technologies has made it feasible to construct precise ad hoc networks [5]. By incorporating the fundamental principles of dynamic and self-adapting networks from Mobile Ad-hoc Networks (MANETs) into the road environment, VANETs have evolved [6], and VANETs are considered one of the key components for future ITS. Each car in a network is equipped with a wireless transceiver that functions as a router for sharing data between neighboring vehicles when no centralized infrastructure is available [7]. Through Peer-to-Peer (P2P) communication, VANETs can enhance end-users traffic efficiency, infotainment, and road safety, especially in hazardous situations.

**Fig 1:**
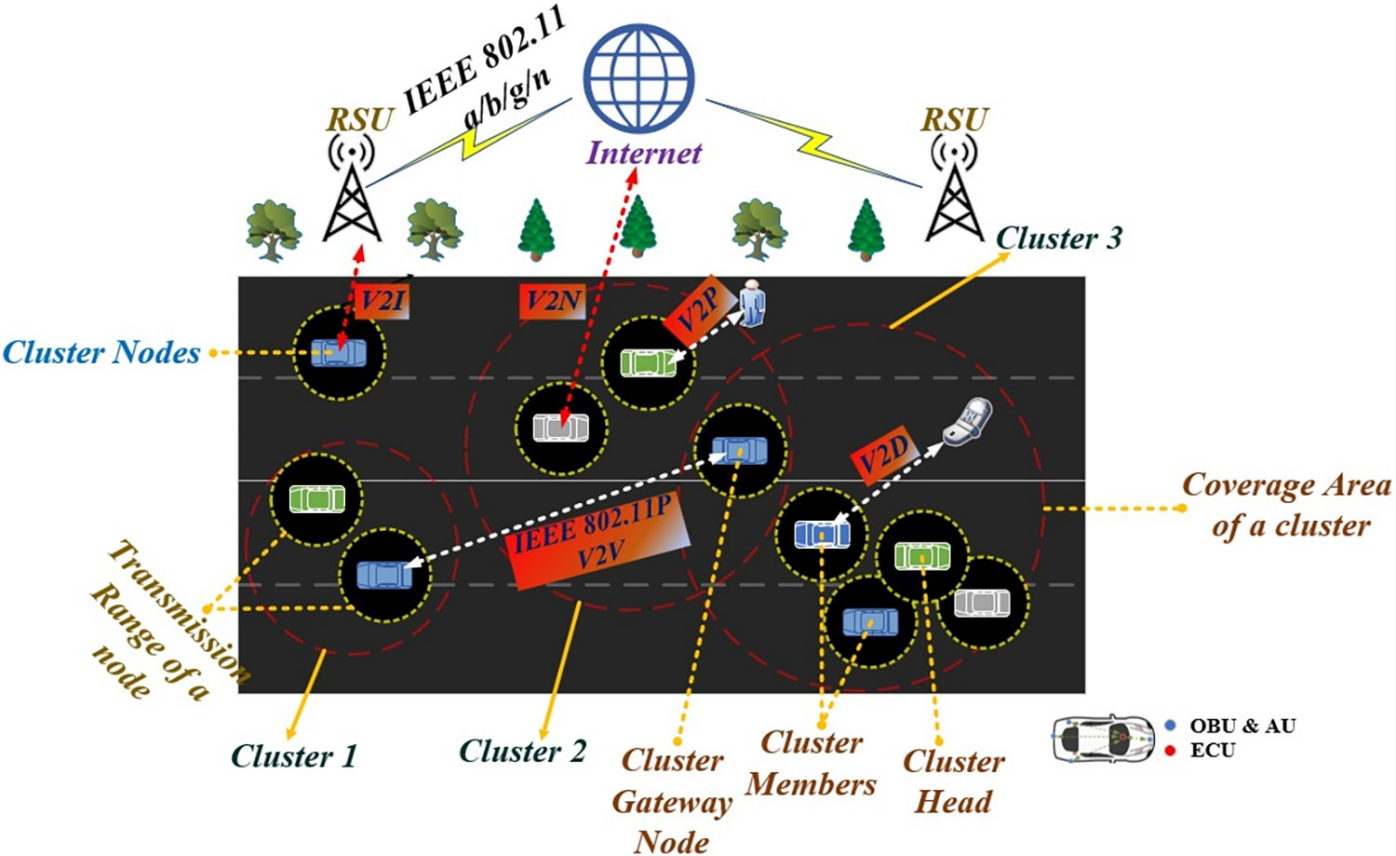
VANET supporting V2X communication by taking advantage of clustering.

VANETs connect with the contrary networks via a global Wireless Access Technology (WAT). To ensure secure and sustainable travel, VANETs have evolved from conventional means of transportation into a network for information gathering and forwarding [8]. This transformation uses the distinguished heterogeneous infrastructures collectively known as V2X communication as shown in Fig 1, which includes Vehicle-to-Infrastructure (V2I), Vehicle-to-Vehicle (V2V), Vehicle-to-Device (V2D), Vehicle-to-Network (V2N), and Vehicle-to-Pedestrian (V2P). WAT is facilitated by DSRC’s next design, IEEE WAVE (Wireless Access in Vehicular Environments), for V2V and V2R, 4G/LTE, as well as Wi-Fi to support V2I, MOST/Wi-Fi to support V2N, and CarPlay/NCF for V2P [4]. On the contrary, several other technologies are available to enable V2X transmission, including Long Term Evolution (LTE), the future 5G, and Cellular V2X (C-V2X). However, the IEEE 802.11p standard is currently the most widely deployed in V2X communication due to its free usage compared to cellular technologies. Furthermore, the IEEE 802.11p standard delivers better delay performance and permits an end-to-end delay of less than 100 ms [9].

In V2X communication, data is exchanged through either single-hop or multi-hop communication. When Line-Of-Sight (LOS) exists among vehicles (typically highway scenarios) with high mobility and relatively low antenna height, a high-power signal is transmitted in single-hop communication [10]. Conversely, the contrary approach (urban scenario) employs minimal transmit power through multi-hop relaying among vehicles and it is generally preferred when no LOS exists between vehicles. The infrastructure of a VANET is constructed with four major considerable components. First, the Application Unit (AU) and On-Board Unit (OBU) are terminal equipment mounted in a vehicle, consisting of distinct modules that can exchange information with Road Side Units (RSUs) and other vehicles within their coverage area. Second, several short-range antennas with fixed infrastructure, in the shape of Road Side Units (RSUs), are installed at strategic geographical points to extend the communication range [11]. RSUs serve as the first point of contact (POC) for OBUs and also provide network services to vehicles, including establishing optimal paths [12]. The network load of RSU depends on the number of vehicles present in the coverage area and it is impossible to monitor all the vehicles which may lead to handoff. Several techniques are present to avoid handoff one such technique is fuzzy logic. One such method is implemented in [13] to reduce handoff and to increase QoS. Third, the Central Control (CC) system is responsible for supervising and monitoring the RSUs already in position. Fourth, data can be shared and broadcast more widely among vehicles and various infrastructures via the Internet [14].

VANETs offer numerous advantages, such as sufficient node energy, powerful data computation, storage capability, a cooperative system, fleet management, and an enhanced navigation system [1]. Nevertheless, VANETs possess distinct characteristics that set them apart from other multi-hop networks. Specifically, (a) high mobility introduces spatiotemporal variations, resulting in an unpredictable network topology that frequently leads to network fragmentation due to frequent link breakages, resulting in message loss. This unpredictable network topology makes VANETs vulnerable to the node problem and leads to high communication overhead due to the constant shifts in vehicle positions and frequent updating in the network [15]. This problem is particularly pronounced in highway scenarios, rendering end-to-end communication. (b) Intermittent connectivity and a sparse distribution of vehicles may result in significant packet loss. (c) VANETs lack centralized management due to their self-organizing nature, making network management a challenging task. (d) Furthermore, VANETs constitute large-scale networks that impose limitations on the communication radius of a vehicle due to limited spectral bandwidth, resulting in poor connectivity among vehicles [16]. (e) Network connectivity can be influenced by non-uniform vehicle distribution and rapid network changes; however, it can be sustained by utilizing a dynamic transmission range instead of a static one [4]. All these issues can profoundly impact scalability, reliability, stability, efficiency, routing, and security, ultimately degrading QoS and End-to-End (E2E) communication. In particular scalability and routing exhibit a direct relationship with the VANET properties and it is represented by using Eq (1).


$${VN}_{scal}\&{VN}_{rout}\;\alpha \;{(V}_{dt},V_{nm},V_{d})$$ (1)

Where **V**_**dt**_ represents the dynamic topology of the network, **V**_**nm**_ represents the node mobility of the vehicle in the VANET environment and **V**_**d**_ represents the vehicle’s density. The authors presented the most recent literature [17] to obtain the best method for finding the optimal path in the VANET environment based on machine learning.

Clustering or platooning is pivotal in VANETs as it acts as the network backbone. Its primary objective is to establish realistic groups of interconnected vehicles that cover all vehicles and road segments within a VANET. MOBIC is a widely used clustering method, but it is restricted to MANETs. The size of a VANET cluster hinges on factors like transmission ranges, grid size, and nodes [18]. Clusters should be formed and maintained to reduce the delay and communication load ratio on the links [19]. Cluster stability, achieved through rules or similarities, enhances network scalability. Key parameters for assessing clustering stability include (a) the number of times the Cluster Head (CH) changes, (b) the duration of the CH, (c) the rate at which Cluster Nodes (CNs) change their CH, and (d) state transitions over the network’s lifetime [20]. While smaller clusters are preferable to longer-sized clusters due to lower maintenance workload and reduced time delays in safety-related messages. Hence Minimizing the cluster generation to near-optimal levels can enhance the stability, and extends the cluster lifetime by contributing maximum time of a nodes to the cluster by which the communication cost can be reduced. Optimal clusters are formed and maintained to reduce the delay and to optimize the load in the network by maximizing resource utilization, and coordination.

### 1.1. The major objectives of the manuscript are as follows

To the best of our knowledge, this is a pioneering effort to implement an intelligent nature-inspired meta-heuristic African Vulture Optimization algorithm on a VANET environment for the first time after observing inherent flaws in state-of-the-art existing approaches, and the proposed work includes the following significant contributions:

- Instead of traditional clustering algorithms, we implemented an Intelligent AVOCA for Clustering in VANETs is mathematically modeled as a MOP and each objective is assigned a self-adjusted weight based on the fitness function.
- The primary objective is to enhance the stability by increasing cluster lifetime in VANETs by optimizing the node clustering by which load optimization can be achieved through effective resource utilization. The local optimum problem is avoided by incorporating AVOCA into the search space.
- The paper explores a taxonomy of the techniques used in clustering, Cluster Head (CH) selection, coordination, and maintenance.

- By comparing the proposed AVOCA technique to existing state-of-the-art approaches, this manuscript improves its performance by providing decision-makers with a set of non-dominated solutions. Based on different network grid sizes, node transmission ranges, and network nodes, the simulation results show that the proposed technique outperforms them.

The remaining article is structured as follows: the 2^nd^ section includes an in-depth literature review based on meta-heuristic clustering; the 3^rd^ section defines the problem statement based on literature. The 4^th^ section elaborates on the state transition that occurs in cluster formation, maintenance, and cluster leaving with pseudocode and flowchart. The 5^th^ section elaborates on the proposed method, which includes a mathematical model as well as pseudocode, the 6^th^ section shows the experimental arrangement, and statistical analysis, and finally results are represented schematically, in 7^th^ section concludes the paper and addresses future work.

## 2. Literature survey

To address VANET-related issues, multiple authors have proposed different strategies based on nature-based meta-heuristic algorithms. one such approach based on the Whale Optimization Algorithm (WOA) was suggested by G. Husnain and S. Anwar in [18] titled, Whale Optimization Algorithm for Clustering in Vehicular Ad hoc Networks (WOACNET). By selecting the optimal cluster, it can optimize the routing, which improves manageability and scalability. The designed WOACNET reduces communication costs in dense traffic environments by leveraging intelligence and capability. Same authors G. Husnain and S. Anwar in [21] propose an extension variant titled Intelligent Probability-based Bio-inspired Whale Optimization Algorithm (i-WOA) that uses a probability-based function and self-adaptive weights to enhance the performance of WOACNET by minimizing network randomness. To avoid exorbitant RSUs and enhance VANET mobility management, the authors S.R.V.Kittusamy et al. in [22] propose a hybrid model that combines adaptive weighted clustering protocol (AWCP) and Enhanced Whale Optimization Algorithm (EWOA). It organizes random nodes by analyzing node movement using Vehicle Network Mobility (VNM) and then archives optimal CH based on mobility and vehicle trust. In [14], the authors O.Senouci et al. suggests a novel heuristic technique for clustering based on RSU called HCAR to address the challenges encountered in IoV. It entails centralizing a clustering algorithm at distributed RSUs, leveraging Graph Theory Concepts for cluster formation, and selecting SCH using a weighted mechanism. To achieve scalability and stability and improve communication efficiency in the IoV topology with local traffic, the authors S.Ebadinezhad et al. suggest another approach, CACOIOV in [4]. The first node is intelligently chosen within the search space, then ACO convergence speed is balanced using the dynamic evaporation rate method, and finally, the DA-TRLD algorithm is executed to maintain IoV network connectivity. Different authors have proposed other promising approaches for enhancing IoV performance through vehicle clustering. The authors A. Salim et al. propose a Swarm optimization-based and mobility-aware clustering method (SOMACA) based on the Sparrow Search algorithm (SSA) [23]. CH is chosen based on SSA, and the optimal link list is sorted from high to low to find the best one. SSA interspersed with GA is another strategy proposed by the authors A.M.Khedr et al. in [24] to enhance effective communication in high mobility nodes titled, Enhanced Sparrow Search Algorithm for IoV (ESSAIoV). Advanced Greedy Hybrid Bio-Inspired (AGHBI) is a distinct approach proposed by the author R.Attia in [25] to address issues and improve performance in IoV. The authors Z.Khan et al. propose another version of ACO with a street-centric routing scheme (SCRS) for Bus-based VANETs [26] that addresses optimal-route along with relay-bus selection for Internet of Energy (IoE) in terms of computational cost and time by reducing unnecessary beacon messages.

In [27], the author Y. Ali Shah introduces AMONET, which employs Moth-Flame Optimization (MFO) to enhance communication efficiency in VANETs through a well-established procedure. AMONET enhances cluster stability by exploring more proficient search spaces using transverse orientation, resulting in optimized clusters. Other MFO variants, ICMFO and CAMONET, are proposed by different authors in [28, 29], respectively. These variants aim to establish efficient clusters to improve communication reliability and stability. Additionally, [28] achieves load balancing and reduces computational complexity. In [5], the author M.M. Hamdi presents an adaptive jumping multi-objective firefly algorithm (AJ-MOFA) along with a clustering and forwarding mechanism (CFM). This combination ensures a high QoS by disseminating data through clustering and eliminating potentially hazardous conditions caused by broadcast storms. The proposed algorithm, referred to as priority-based data dissemination (CPDD), can discover more dominant solutions through objective decomposition, archive management, and a mutation-based trade-off between the exploration and exploitation phases. In [30] the authors A. Zeynivand et al. improves the QoS for inter-road communication through traffic control for optimizing travel times, and queue lengths by using multi- agent reinforcement learning. Furthermore, the author C.J. Joshua introduces another variant in [31], where the Reputation-based Weighted Clustering Protocol (RWCP) stabilizes VANET topology without introducing overhead. To optimize RWCP, the Multi-Objective Firefly Algorithm (MOFA) is utilized. In [32], the authors R. Dhanare et al. propose a hybrid approach called Modified Ant Colony and Firefly Optimization Techniques (MAF) to address the issue of computing average speed through clustering during catastrophic events. The authors C.S. Evangeline and Vinoth Babu k in [33] improve the QoS of the VANET based on the two-phase access network selection. In the first phase, available networks are ranked based on demands using the weight sum method and in the second phase network selection is carried out using the game theory approach.

Various meta-heuristic algorithms have been developed to address security, overhead, and performance issues and enhance V2V and V2I communication in VANETs. In [32], the authors S. Sharma and A.Kaul utilize a Hybrid Fuzzy Multi-Criteria Decision-making Approach (HF-MCDM) to establish resilient multiple CH, complemented by the Dolphin Swarm Algorithm for intrusion detection. In [8], the authors X.Bao et al. employ Particle Swarm Optimization (PSO) not only for defining CH but also for route optimization within VANETs. In [34] the authors S.A.Javadpour et al. strengthens road protection in VANETs by detecting unpredictable problems and replacing broken paths with immediate effect based on Quality of Service Routing (QoSR) and Particle Swarm Optimization (QoSR-PSO) information.

In [35], authors S.Hosmani and B.Mathapati focus shifts to Robust and Reliable Secure Clustering and Data Transmission (R2 SCDT), which is based on trust values, providing secure and reliable communication. The authors A. Ali et al. in [36] introduce the Harris Hawks Optimization (HHO) algorithm to address network fragmentation, scalability, overhead, and packet routing issues, to improve the performance effectively. In [19], the authors M.Ahmad et al. propose the Vehicular Genetic Bee Clustering (VGBC) approach, which minimizes the size of routing tables through clustering, ultimately reducing overhead in the system. To further enhance system efficiency and maximize transmission rates in urban VANETs, authors L.Sellami and B.Alaya in [37] present the Self-Adaptive Multi-Kernel Clustering for Urban VANET (SAMNET). This approach relies on collecting measurement data generated by linear sub-models that communicate via unpredictable dynamic switching. In [15], the authors Z.Yang et al. introduce a novel method using the Route Time function to identify overlapping periods among vehicles based on navigation route information. This approach helps maintain vehicles as neighbors along their routes, improving network stability. In [38], authors R. Regin and T. Menakadevi suggest another approach called Density-Based Dynamic Clustering (DBDC). DBDC leverages precise location data to minimize network overhead and proactively address congestion. By setting a vehicle density threshold, DBDC prevents network delays and packet loss. In [39], the authors M. Mukhtaruzzaman and M.Atiquzzaman focus on Junction-Based Clustering for VANETs (JCV). They emphasize the importance of the moving strategy at preceding junctions and consider various parameters during cluster creation to enhance stability. In [40], the authors D. Zhang et al. propose Passive Multi-Hop Clustering (PMC) to establish inter-cluster nodes, prioritizing stability and reliability over node mobility. The approach employs a priority-based neighbour-following strategy, optimally selecting neighbours, and employs a cluster merging mechanism to enhance cluster coverage while reducing interference.

A review of the literature on different methods for clustering to optimize the performance in VANETs provides several prominent pros and cons of state-of-the-art algorithms as shown in Table 1. However, due to the dynamic and unpredictable nature of VANETs, current state-of-the-art algorithms have several limitations. As a result, there is still plenty of space to optimize the clustering process to improve overall network performance.

**Table 1:** Some recent state-of-the-art approaches for cluster optimization.

Ref	Approach	Advantage	Disadvantage	Simulator	Future Scope
[22]	AWCP+ EWOA	Clustering efficiency. Mobility enhancement. Vehicle reliability.	The urban scenario is not considered. Sensitive to user parameters.	NS-3	Enhanced mobility management with minimized cost and maximized CE.
[14]	HCAR	Centralized clustering. Improves network control and overhead. One hop clustering.	The urban scenario is not considered. Early convergence	NS-2 and Vanet MobiSim	HACR in urban areas.
[4]	ACO +DA-TRLD	5G interfacing. Avoids network dissemination. Cluster-based optimization	The urban scenario is not considered. Local optima. Sensitive to user-defined parameters.	NS-2	D2D communication. To extend beyond 5G.
[27]	AMONET	Effectively work in high mobility nodes. Low cost. Reduced packet delay. Minimum hops	Local optima. The urban scenario is not considered.	---	Node re-affiliation frequency can be calculated. Tx range and No. of nodes can be changed.
[5]	AJMOFA +CFM	Contains 3 phases	The highway scenario is not considered. Local optima.	MATLAB	Data dissemination in various scenarios. Hyper-heuristic-based parameter selection.
[15]	Navigation Route Inf	Achieves stability. Reduce communication costs.	Scenarios are not considered.	Ns-3	CH election based on intelligence Optimizing cluster size.
[37]	SAMNET	Random and continuous traffic. Contains 3 phases. Modeling based on linear regression. Balanced load.	The highway scenario is not considered.	MATLAB	Implementing in more complex real-time scenarios. Implementing M.L. to optimize the SAMNET.
[18]	WOAC- NET	Route optimization	The urban scenario is not considered. Early convergence.	---	Implementation for multi-objective functions and rapidly changing topologies.
[41]	HF-MCDM	Integration of two phases. Solve intrusion detection. Low CH load and delays.	Low dense network scenario is not considered.	Netsim and MATLAB	Implementation of proactive mechanism in a cloud-based network.
[29]	CAMO- NET	Values of objective function modified. Greater search space. Low routing cost.	The urban scenario is not considered. Unpredictable dynamic topology. Cluster instability	MATLAB	Nodes can be set to dynamic. Enhance the list of objectives.
[36]	HHO	Maintains equilibrium state between phases. Low Resource consumption. Reduced no. of hops. Low latency.	Sensitive to user-defined parameters. Unpredictable dynamic topology. Computationally Expensive	MATLAB	Implantation in 5G. Implementation for FANETs. Optimal resource allocation. More objectives according to the network environment.

## 3. Problem statement

### 3.1. Node clustering as a problem of optimization

The quantity and complexity of real-world optimization problems in AI are increasing every day, and they have become significant in scientific, engineering, and decision-making applications. Optimization problems may encompass continuous, discrete, nonlinear, multi-model, and dimensional, often referred to as multi-objective problems (MOPs). These characteristics challenge traditional mathematical optimization paradigms, such as the Quasi-Newton method and Quadratic Programming. Researchers have also demonstrated that such techniques typically yield only a single solution in a single run, which is insufficient for solving MOPs [36]. Some Evolutionary Algorithms (EAs) based on nature-inspired have been proposed as competitive alternative solvers for addressing real-world MOPs. Nature-inspired algorithms can generate a set of solutions, often referred to as optimal solutions, in a single run as shown in Eq (2) [42].


$$Z=X_{1}(Y_{1}(d))+X_{2}(Y_{2}(d))+.\ldots +X_{n}(Y_{n}(d))$$ (2)

The final value of “Z" can be calculated based on the weighted objective function, where X_i_ is the i^th^ objective function’s weight ranges from [0 1], and d represents the decision variable.

Examples of EAs include Genetic Algorithms, Evolutionary Strategies, meta-heuristic approaches, and Learning Classifier Systems. While EAs are computationally expensive, they excel at finding fast optimal solutions, and effective choices for problems that are challenging to solve using other techniques [12]. Furthermore, harnessing the computational power of hardware and incorporating stochastic operators enhances their strength and effectiveness in exploring the search space for optimal solutions [5]. The implementation of EA for solving NP-hard (non-deterministic polynomial-time hard) problems represents a class of computational challenges for which finding an optimal solution in polynomial time is considered impractical as the size of the network increases, with the Traveling Salesman Problem (TSP) serving as a quintessential example. The complexity of solving particular problems like optimal data aggregation, optimal nodes for data dissemination, optimal routing, and optimal node clustering falls into the NP-hard category in VANETs. For instance, considering N vehicles, the number of possible routes to explore is N!. In real-world dynamic networks, as N increases, this factorial growth renders an exhaustive search computationally infeasible. To Address NP-hard problems within reasonable time frames for enhancing reliability and scalability, the development of heuristic algorithms becomes a significant concern and it is proven to be highly effective.

## 4. Clustering transition stages in VANETs

According to the No Free Lunch (NFL) theorem [38], a single approach cannot handle all optimization problems at the same time. As a result, an optimizer might generate adequate results in one scenario but fail in another scenario. Hence African Vulture Optimization approach is proposed for optimal clustering and CH formation for the VANET environment. The proposed algorithm is initiated with the exploration phase, followed by the exploitation phase, once every vehicle successfully registers in the network. The selection of CH is based on the fitness function of each vehicle.

### 4.1. Traffic generator

Initially Probability Density Function (PDF) is used to generate N vehicles in the highway scenario and their speed follows a Gaussian Distribution **G**_**pdf**_(**N**), as shown in Eq (3). The speed difference between two neighboring vehicles is calculated as shown in Eq (4). Eq (5) is another PDF used to generate the time interval among the batches and follows an Exponential Distribution [43].


$$G_{pdf}(N)=\frac{1}{\sigma \sqrt{2\pi }}e^{\frac{{-(N-\mu )}^{2}}{{2\sigma }^{2}}}$$ (3)

Where σ represents the standard deviation i.e., the spread of vehicle speed around the mean, and **μ** is the average speed of highway vehicles.


$$G_{pdf}(\Delta N)=\frac{1}{\sigma \Delta N\sqrt{2\pi }}e^{\frac{{-(\Delta N-\mu \Delta N)}^{2}}{{2\sigma \Delta N}^{2}}}$$ (4)

$$E_{pdf}(T)=\left\{ \begin{array}{l} {\lambda e}^{-\lambda T},\;T\geq 0\\ 0,\;T<0 \end{array}\right.$$ (5)

Where Δ**N** = **N**_**1**_−**N**_**2**_ and **μΔN** = **μ**_**1**_−**μ**_**2**_, T denotes the time interval and $\frac{1}{\lambda }$ denotes the expected time interval between two consecutive batches.

Each vehicle is assigned a specific acceleration with random variables R1 and R2 by using Eq (6). Meanwhile, acceleration is controlled by [**acc**_**i**_ **P**_**r**_] while deceleration is regulated by [**dacc**_**i**_ **P**_**r**_] with both parameters influenced by the Aggressiveness of Driving Behavior (AGG). The R2 goal is to give the vehicle a random value of acceleration within [0, Amax] or deacceleration within [Dmax, 0], whereas the R1 goal is to give the vehicle one of three decisions (acceleration, deacceleration, or neither). Eq (6) is integrated by Eq (7) to obtain the velocity of a vehicle, which is the Gauss-Markov Model, where t denotes the time and i represents the vehicle index. Distance is obtained by integrating Eq (8) with Eq (7).


$$a_{i}(t)=\left\{ \begin{array}{l} R_{2}.A_{max},\;if\;\;R_{1}<{acc}_{i}+P_{r}\\ R_{2}.(-1).D_{max},\;if\;{acc}_{i}+P_{r}\leq R_{1}<{acc}_{i}\\ \;+{dacc}_{i}+2_{P_{r}}\\ 0,\;Otherwise\\ \; \end{array}\right.$$ (6)

$$V_{\psi }(t+\Delta t)=V_{\psi }(t)+a_{i}(t).\Delta t$$ (7)

$$X_{i}(t+\Delta t)=X_{i}(t)+V_{i}(t).\Delta t(5)$$ (8)

#### 4.1.1. Feature extraction

Feature extractions are classified into network features (vehicle ID features). The second category pertains to mobility, defined by three variables for each vehicle and their inertial frame x and y-axis projections. Specifically, **X**_**i**_ **and Y**_**i**_ define the position, **V**_**ψ(x)**_**(i) and V**_**ψ(y)**_**(i)** define the velocity, and **a**_**x**_**(i) and a**_**y**_**(i)** define the acceleration. The relationship between these components and the body frame is depicted in Eq (9).


$$F_{E}(i)=R(\theta ){\left(0,0,V_{\psi (xb)}(I),V_{\psi (yb)}(I),a_{xb}(I),a_{yb}(I)\right)}_{\;}^{T}+Trans(x_{gps}(i),y_{gps}(i))$$ (9)

Where **x**_**gps**_**(i), y**_**gps**_**(i)** represents the vehicle’s GPS coordinates and **θ** denotes the angle between the vehicle on the road and the inertial frame.

### 4.2. Neighbourhood exploration & cluster formation

**Fig 2:**
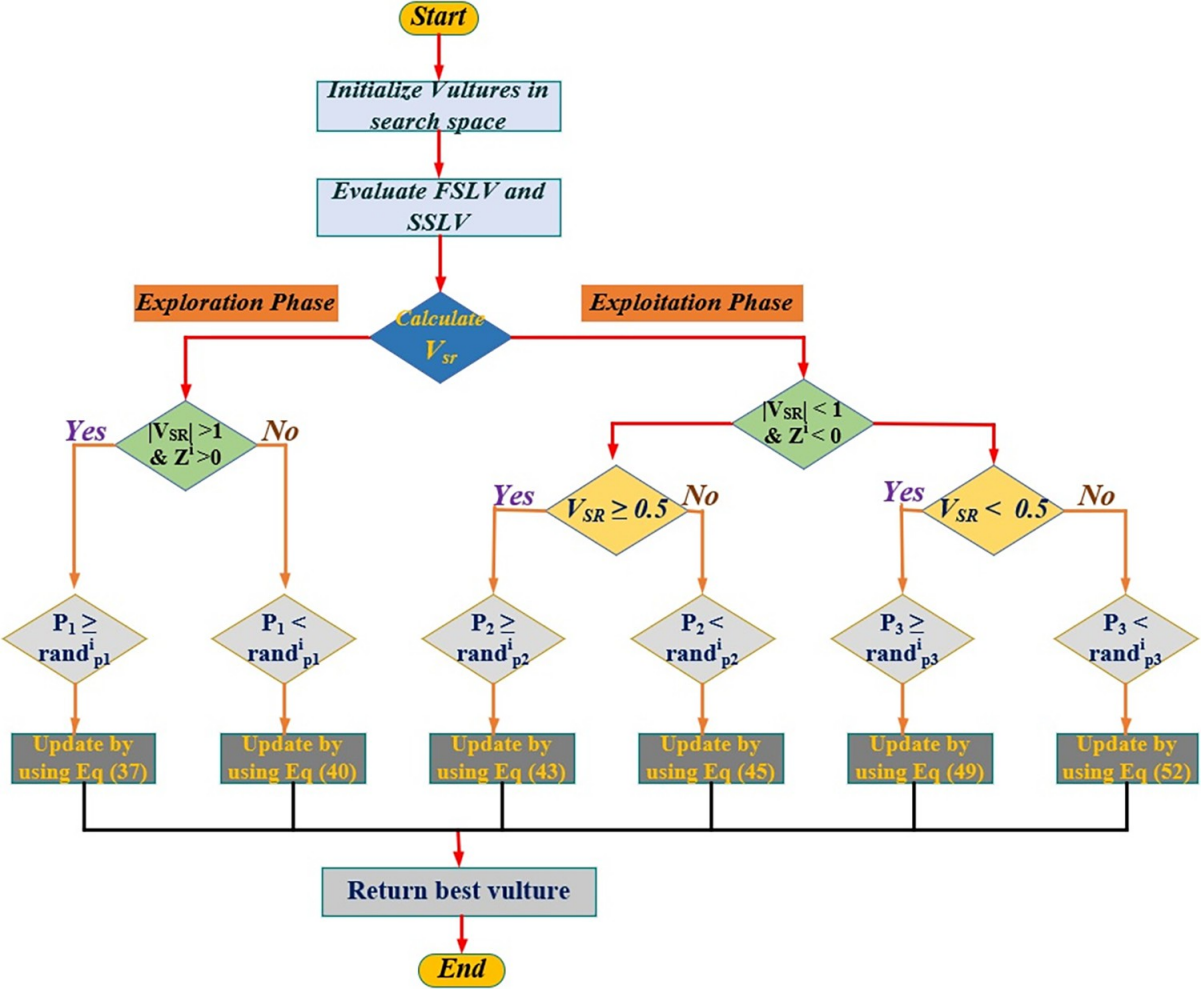
General procedural flow of clustering algorithm.

As VANETs are dynamic, a vehicle may enter or leave a cluster at any time if it is a member of a neighboring node. When a vehicle ’V’ initially gets to the road, it is in the **VN**_**un**_ state. Once the vehicle decides to join the network, its communications system is activated. Initially, the node operates as a member, broadcasting periodic **BC**_**msg**_ as **H**_**msg**_ while simultaneously gathering identical data from its n-hop neighbors. The node starts a timer to search the existing cluster by broadcasting **J**_**REQ**_, and activate a flag to represent the arbitration process. Meanwhile, it can communicate with the RSU within its communication range. Within **TP**_**n**_, if a node receives a **J**_**REP**_ response from an existing cluster, it participates in the cluster as a **VN**_**cm**_. However, if the node receives responses from multiple clusters, it will join the cluster with the highest priority. If a node does not receive a response from the existing clusters within a **TP**, the node initiates the cluster formation process by broadcasting itself as a **VN**_**ch**_ and forming its own **VN**_**c**_ and pseudocode is shown in Table 2. If a **VN**_**ch**_ wishes to change its status to **VN**_**cm**_ or leave the cluster, it must delegate its responsibilities to the **VN**_**sch**_. If two clusters decide to merge, any of their **VN**_**ch**_ statuses may be changed to **VN**_**cm**_ or **VN**_**sch**_ of the merged cluster. If a node is no longer the **VN**_**cm**_ of the current cluster, it can change its status back to **VN**_**un**_ or leave the network. Vehicles generally establish neighborhood relationships by embedding current **V**_**p**_ & **V**_**ψ**_ into **H**_**msg**_ and broadcasting within their communication range. A primitive group is made up of vehicles that proceed in the same direction and are near one another. However, speed levels may differ between locations, and this deviation can be significant. Consequently, not all neighboring vehicles are eligible for inclusion in a single cluster. The formation and ongoing execution of clusters involve several key steps that must be repeated based on the algorithm’s standards and the network’s mobility behavior. The general procedural flow of a clustering algorithm is depicted in Fig 2 and the most commonly used notations are specified in Table 3.

**Table 2:** Optimal cluster formation approach.

**Algorithm:** Pseudocode for cluster formation
**Input:** Set of individual vehicles V = {V_0_, V_1_, V_2_……V_n−1_}
1. Vehicle enters into the network
2. **If**
3. State of vehicle → *VN*_*un*_
4. Node initially acts as *VN*_*cm*_ **&** broadcast *BC*_*msg*_ as H_msg_
5. Node initiates time by J_REQ_ **&** Node.flag = 1
6. **While (**Not receive *J*_*REP*_ **OR** timer = = 0)
7. Wait
8. **If** (not receive *J*_*REP*_ **OR** timer = 0)
9. Repeat from step 4 again
10. **Else**
11. Initiates the cluster formation to identify VN_cm_
12. Broadcast itself as a VN_ch_ and form its own *VN*_*c*_
13. **End If**
14. **End While**
15. **If** (receive *J*_*REP*_ **&** timer = = 0) **OR** (receive multiple *J*_*REP*_ **&** timer = = 0)
16. Node flag = 0
17. Change its status to *VN*_*cm*_ by joining
18. **End If**
19. **If** (*VN*_*ch*_ change status to *VN*_*cm*_ **OR** leave cluster)
20. *VN*_*ch*_ responsibilities are given to *VN*_*sch*_
21. **End If**
22. **If** (two clusters merge into a single cluster)
23. *VN*_*ch*_→*VN*_*cm*_ **OR** *VN*_*sch*_ **OR** *VN*_*un*_
24. **End If**
25. **End If**
**Output:** Clustered vehicles C = {C_1_, C_2_, C_3_,……C_n_}

**Table 3:** Most commonly used notations in the clustering stage.

Notation	Description	Notation	Description
ID_v_	Identity of Vehicle (V)	V_fd_	Vehicle Forward Direction
V_tr_	Tx range of a Vehicle (V)	V_bd_	Vehicle Backward Direction
V_p_	Position of a Vehicle (V)	VN_un_	Undefined Node
V_ψ_	Velocity of a Vehicle (V)	VN_c_	Cluster in VANET
TP_n_	The period of a node	VN_ch_	Cluster Head
BC_msg_	Beacon Message	VN_sch_	Secondary Cluster Head
H_msg_	Hello Message	VN_cm_	Cluster Member
J_REQ_	Join Request	VN_cg_	Cluster Gateway
J_REP_	Join Reply	C_cm_	Members List Table in Cluster
TH_v_	Threshold Value	Tx	Transmission Range

#### 4.2.1. To find the optimal no. of clusters

Determining the optimal no. of clusters is a critical task in VANETs to achieve load optimization by ensuring that the available resources are used effectively or allocated which is represented by Eq (13). Balancing the workload among the vehicles in the clusters to prevent congestion is another way to achieve load optimization which leads to enhanced network stability. Load balancing among vehicles is calculated by using Eq (11). Optimal routing can also balance the load optimization in the network and it is calculated by using Eq (12). Load optimization can also be achieved through maintaining power constraint which is represented by using Eq (14). Eq (10) is used to calculate the load optimization which minimizes the communication overhead and reduces latency in the network.


$$f(w,x,y,z)=w_{1}.V_{lb}(w)+w_{2}.V_{rc}(x)+w_{3}.V_{ru}(y)+w_{4}.V_{pc}(z)$$ (10)

$$V_{load\;balancing}(w)=|\frac{1}{n}\sum_{i=1}^{n}x_{i}-target\;load|$$ (11)

$$V_{routing\;cost}(x)=\sum_{i=1}^{n}\sum_{j=1}^{n}y_{ij}.{cost}_{ij}$$ (12)

$$V_{resource\;utilization}(y)=\sum_{k=1}^{m}z_{k}$$ (13)

$$V_{power\;constraint}(z)=P_{i}\leq P_{max}$$ (14)

Where *w*_1_, *w*_2_, *w*_3_ are weights assigned to each component, *y*_*ij*_ is a binary variable indicating a direct link between clusters i and j and *cost*_*ij*_ represent the cost associated with the link, *P*_*i*_ represents the transmission power for vehicle I and *P*_*max*_ represents the maximum allowable power.

To calculate the no. of clusters formed, we begin by calculating the radius of a circle referred to as the Vehicle Range (VR). Each cluster is designed to cover the maximum possible area within the VR in m^2^. Eq (15) calculates the vehicle connectivity percentage within a circular area. Subsequently, utilizing the vehicle thickness as outlined in Eq (16), we establish connectivity among all cluster members.


$$VR=\pi .r^{2}$$ (15)

$$VT=\frac{v}{y}$$ (16)

$${VN}_{c}=|VR.VT|$$ (17)

Where v and y are used to represent the total no. of vehicles and height of the network. For example, if v is 200 vehicles and y is 7500 then the outcome of VT is 0.026666. VR and VT have further been used to compute **VN**_**c**_ as shown in Eq (17) which represents the maximum number of vehicles that become part of one cluster. Considering the output of VT is 0.026666 and VR is 3140 (where ***r***^**2**^ = 1000 and π = 3.14), then the absolute value of **VN**_**c**_ is 83. It means a maximum of 83 vehicles become part of one cluster.

#### 4.2.2. To find cluster lifetime

The cluster lifetime in VANETs refers to the duration for which a specific cluster configuration remains active and functional before undergoing any changes or dissolving. Eq (18) is used to calculate the lifetime of a cluster. Where **V**_**m**_ denotes the vehicle mobility on the highway and it is calculated using Eq (19). **V**_**cr**_ and **V**_**tp**_ represents the vehicle’s communication range and vehicle Transmission power which is calculated by using Eq (20). **V**_**d**_ represents the number of vehicles per unit length of the highway and it can be calculated using Eq (21).


$${VN}_{CL}=f(V_{m},V_{cr},V_{d},V_{tp})$$ (18)

$$V_{m}=\frac{{\Delta V}_{position}}{{\Delta V}_{time}}$$ (19)

$$P_{r}=P_{t}(\frac{G_{t}G_{r}\lambda^{2}}{{\left(4\pi d\right)}^{2}l})$$ (20)

$$V_{d}=\frac{N}{L}$$ (21)

Where ΔV_position_ represents the change in the vehicle position for a change in time ΔV_time_, P_r_ and P_t_ represents the transmitted and received power, G_t_ and G_r_ represent the gains of the transmitting and receiving antennas, λ represents the wavelength of the signal, l represents the system loss factor, d is the distance between the transmitter and the receiver, N represents the number of vehicles on the highway and L represents the length under consideration.

### 4.3. Cluster head formation

The careful selection of CH is crucial to minimize communication overhead calculated by using Eq (25), which refers to the extra data or signaling beyond the actual content of the messages involved in communication and extends the lifetime. Traditionally, factors like speed and direction, distance, mobility, stability, and density have been used for CH selection. However, in this approach, CH selection is based on the vehicle’s trust value calculated by using Eq (22) and the location of a vehicle. Vehicles with a high trust value have a better chance of becoming a CH as shown in Table 4. Each vehicle needs to determine whether both directions—front and rear—are full, empty, or semi-full of neighbors based on the condition given in Eq (23). This information helps a vehicle decide whether to participate in the CH selection. For instance, a vehicle positioned at the tail or front of a cluster lacks one-hop neighbor communication and should not be considered a viable CH candidate. Ideally, a CH should be located at the cluster’s center as shown in Eq (24), as it’s better equipped to manage the cluster efficiently. It means the CH should have an equal or roughly similar number of neighbors in both directions, ensuring balanced communication and effective cluster management.


$$TV=\frac{\left((W_{1}x{TF}_{1})+(W_{2}x{TF}_{2)}+(W_{3}x{TF}_{3})\right)}{3}$$ (22)

$$\left\{ \begin{array}{l} V_{fd}=0\;OR\;V_{bd}=0\;(semi)\\ \;V_{fd}\;\;V_{bd}>0\;(full)\\ \;V_{fd}=0\;V_{bd}=0\;(null) \end{array}\right.$$ (23)

$$V_{fd}≅V_{bd}\;(condition)$$ (24)

$${VN}_{comm\;overhead}=\frac{\sum_{i=1}^{N}\left(V_{msg\;freq}+V_{Msg\;Size}\right)}{Total\;no.of\;vehicles}$$ (25)

$$V_{msg\;freq}=\frac{n}{T}$$ (26)

$$V_{Msg\;Size}=(Header\;Size+payload\;size+Additional\;Information)$$ (27)

Where, **V**_**Msg Freq**_ represents the rate at which messages are transmitted per unit of time i.e. messages per second and it is mathematically represented using Eq (26). **V**_**Msg Size**_ denotes the size of each message transmitted or received which can be represented by using Eq (27). n represents the number of messages transmitted or received by the vehicle and T represents the total time.

**Table 4:** Optimal cluster head approach.

**Algorithm:** Pseudocode for cluster head formation
**Input:** Set of Clustered vehicles C = {C_1_, C_2_, C_3_,……C_n_}
1. **For** all VN_cm_ collects and monitors neighborhood information
2. **If** V_p_ is (V_fd_ = 0 & V_bd_ = 0)
3. Then no chance to participate
4. **End If**
5. **If** V_p_ is ((V_fd_ = 0 **OR** V_bd_ = 0) **OR** (V_fd_ & V_bd_>0))
6. Then VN_cm_ has a chance to participate as VN_ch_
7. Set VN_ch_flag_ = True
8. **End If**
9. **For** each vehicle in the cluster
10. Compute the Trust valve and broadcast to all groups within the network
12. **If** (V_fd_≅V_bd_) **&** Trust valve > all vehicles in cluster
13. then VN_cm_ can declare as VN_ch_ and updated through broadcast
15. **Else**
16. VN_cm_ will be member
17. **End If**
18. **End For**
19. **End For**
**Output**: VN_ch_ for individual cluster

### 4.4. Cluster maintenance

#### 4.4.1. As a cluster head

CH is responsible for cluster formation and termination, data transmission, cluster interface, relaying functions, topology selection, and distributing resources to CM. Since CH changes frequently due to the dynamic nature of VANETs, reducing the total number of CHs is preferable. CH handles communication within the cluster that is more than one hop away, between clusters, and between nodes and the RSU.

#### 4.4.2. As a cluster member

The node will check its link to its CH regularly by expecting a poll frame sent by the CH or by actively sending "alive" messages. If a node’s connection to its CH fails, it may exit the cluster and join another cluster as a new CM, or it may create its own cluster. If a node finds an affiliation request by an unclustered node, it can leave its parent cluster and become a CH.

#### 4.4.3. Cluster gateway

CH immediately selects the CGW based on position when the cluster formation phase is complete. Normally, the CM at the head or tail of the clusters is preferred. Hence, it would be the CM of two clusters. These CGW are used to communicate between two CHs. For example, there are three CHs: CH1, CH2, and CH3. The two gateways are CGW1 and CGW2, which are members of CH1 and CH2. CGW3 is the gateway that is present in both CH2 and CH3. When CH1 sends a message, it is the gateway’s responsibility to deliver it to CH2. If any existing CGW moves out of the transmission range, another CGW is automatically selected and updated within the network.

#### 4.4.4. Leaving a cluster

Vehicles on the highway may switch between clusters multiple times. After the cluster formation phase concludes, each CH initiates a monitoring process for its members, maintaining an up-to-date table to track their presence within the cluster. To achieve this, every cluster member regularly sends beacon messages to its CH. CHs employ an intra-cluster gathering process to collect these beacon messages from their CM, allowing them to monitor the presence of CMs within the cluster effectively. Consequently, when a CM leaves the cluster range, the CH detects the event promptly removes the CM from its table, and updates it in the network as shown in Table 5. Furthermore, suppose a CM fails to receive the periodic message from its CH within a specified period then, its state changes to UN (unreachable), and it becomes eligible to join another cluster. When a cluster loses its members, it is considered to have dissolved or "died."

**Table 5:** Cluster leaving approach.

**Algorithm:** Pseudocode for leaving a cluster
1. **If** VN_c_ & VN_ch_ is created
2. VN_ch_ monitors VN_cm_
3. VN_cm_ sends BC_msg_ to VN_ch_, vice versa periodically
4. **If** (VN_cm_ moves > than V_tr_) **OR** (no BC_msg_ received)
5. VN_cm_ deleted from the list and updated within the network
6. **End If**
7. **End If**

#### 4.4.5. Joining a cluster

Several UN attempts to join the network and these are either newcomers or have left other clusters. When a UN vehicle enters the transmission range of a CH, it transmits a beacon message containing its information. If the UN vehicle’s information matches the CH’s, it is welcomed into the cluster and the vehicle state is changed from UN to CM. The CH adds the new vehicle to its members list and updates the network accordingly. In cases where there’s no match between the UN vehicle and the CH, the CH ignores the request, the pseudo-code is shown in Table 6.

**Table 6:** Cluster joining approach.

**Algorithm:** Pseudocode for joining a cluster
1. **If** VN_c_ & VN_ch_ is created
2. several VN_un_ seek to join by sending BC_msg_
3. **If** VN_ch_ data = VN_un_
4. then VN_ch_ accepts to join with ACK
5. updated the table and within the network
6. **Else**
7. simply ignores VN_un_
8. **End If**
9. **End If**

#### 4.4.6. Cluster head leaving at SCH

A secure communication link is established between the CH and SCH, they engage in regular message exchanges to update their status. Consequently, the CH continuously monitors the SCH’s status and vice versa. If the SCH stops receiving messages from its CH within the expected timeframe, it signifies that the CH has left the cluster, necessitating it to take over its role. Similarly, if the CH ceases to receive messages from the SCH, the network must be updated by designating the old SCH and appointing a new SCH. This update is accomplished by transmitting beacon messages within the network and the pseudocode is shown in Table 7.

**Table 7:** CH leaving at SCH approach.

**Algorithm:** Pseudocode for SCH
1. **If** VN_c_ & VN_ch_ is created
2. VN_sch_ is chosen within the cluster
3. If BC_msg_ receives periodically within TP
4. VN_ch_ & VN_sch_ will be continued
5. **Else**
6. new (VN_ch_ **OR** VN_sch_) is selected
7. updated their status and within the cluster
8. **End If**

### 4.5. Cluster MERGING

The merging aims to prevent overlapping between two clusters close to each other, resulting in interference. Thus, to merge the CHs, the distance and transmission range between them are calculated and compared to a pre-defined value. The CH with the lowest suitability value relinquishes its CH role and joins the other cluster. The old and new CH, SCH, and CM are then updated in the table, along with the Neighbourhood, the pseudocode is shown in Table 8.

**Table 8:** Cluster merging approach.

**Algorithm:** Pseudocode for cluster merging
1. **For** instance VN_c1_ & VN_c2_ is created
2. **If** (VN_c1_(*T*_*r*_)≈VN_c2_(*T*_*r*_))
3. merge VN_c1_ & VN_c2_
4. A new cluster is formed
5. new (VN_ch_ & VN_sch_ & VN_*cm*_) are selected
6. updated their status and with the Neighbourhood
7. **End If**
8. **End If**

### 4.6. Hidden node challenges

The VANET environment encounters hidden node issues during one-hop communication, where a node is not directly within the transmission range or line of sight of another node. This lack of direct awareness can result in interference and collisions when multiple nodes attempt simultaneous transmission, causing communication breakdowns and packet loss, posing challenges to reliability. Once after clustering, CH actively tries to find the presence of hidden nodes. Once it is detected, the adjustment in transmission power is done, and data is retransmitted. Conversely, if congestion surpasses the threshold or the transmitted signal exceeds the specified time, indicating message loss, the data must be retransmitted from the beginning. The systematic steps taken to mitigate hidden node is represented through pseudocode in Table 9.

**Table 9:** To mitigate hidden node challenges.

Algorithm: Pseudocode to mitigate hidden node challenges in VANET environment
1. Input: Existing hidden node terminals in the VANET environment
2. Initialize the VANET parameters
3. While vehicles enter into the VANET environment do
4. If vehicles exhibit similar parameters
5. Join them to form a new cluster
6. Else
7. Add to the existing cluster
8. End If
9. End While
10. If
11. If CH detects the hidden nodes
12. Adjust transmission power
13. Explicit signaling
14. Else
15. Continue with existing parameters
16. Transmit data
17. End If
18. Else
19. If (congestion > TH_v_) OR (TX_signal_>T_p_)
20. Message is lost
21. Retransmit the data
22. Else
23. Continue with the transmission
24. End if
25. End if
26. Output: Mitigates hidden node challenges

## 5. Proposed cluster optimization in VANETs using African vulture optimization algorithm

### 5.1. The biological life of African vultures

African vultures are a species native to the African continent, and they play a crucial role in both ecosystems and human societies. Most vultures are bald, which is an adaptation that helps them avoid contamination and stinging while feeding on carcasses, particularly in tropical regions. Distinguishing features that set vultures apart from most other birds include keen vision, conservation challenges, migratory behavior, cultural significance, and a long lifespan. Vultures are typically classified into three types based on their agility. Vultures’ tendency to spend hours searching for food leads them to travel long distances using rotational flight. Sometimes, when all vultures converge on a single food source rather than individual ones, conflicts can arise among them [44].

### 5.2. African vultures optimization algorithm

In a given environment, the population size consists of approximately N vultures, and it can vary according to the researchers’ problem requirements. Initially, the algorithm calculates the fitness function for all individuals in the initial population, grouping vultures into categories. During the formulation phase, our anti-hunger principles lead us to believe that the worst solution within the population is the most fragile and hungry. The AVOCA algorithm considers the two best solutions as the strongest vultures compared to others within the population. Therefore, vultures strive to distance themselves from the worst solution and aim to converge on the best solution. Based on these fundamental vulture-inspired concepts, the AVOCA algorithm is developed in four distinct phases for simulation, and each phase is comprehensively outlined.

### 5.3. First phase: Determining the best vulture in the population

The optimization process generates non-dominated random solution vectors across the population, which can be mathematically represented as a two-dimensional matrix, as shown in Eq (28). In each iteration, the fitness of the entire population is calculated before the search operation, and two sets of social leaders, namely Social Leader Vultures (FSLV) and Second-generation Social Leader Vultures (SSLV), are selected. These leaders steer the other vultures within the population, as depicted in Eq (29). All non-dominated solutions are included in the FSLV set, from which the best social leader is chosen based on diversity and convergence measurements.


$${AV}_{p}=\left[\begin{array}{ccccc} W_{1,1,} & W_{1.2} & \cdots & W_{1,d-1} & W_{1,d}\\ W_{2,1} & W_{2,2,} & \cdots & W_{2,d-1} & W_{2,d}\\ \vdots & \vdots & \cdots & \vdots & \vdots \\ W_{N,1} & W_{N,2} & \cdots & W_{N,d-1} & W_{N,d} \end{array}\right]$$ (28)

Each row $W_{i}=(W_{i,1},W_{i,2},\ldots \;\ldots \;\ldots ,W_{1,d-1},W_{i,1})$ indicates the African vulture at the ith position.


$$R(i)=\left\{ \begin{array}{c} {BV}_{1}\;if\;{(P}_{i}=X_{1})\\ {BV}_{2}\;if\;{(P}_{i}=X_{2}) \end{array}\right.$$ (29)

$$P_{i}=\frac{V_{sr(w)}^{i}}{\sum_{i=1}^{n}V_{sr(w)}^{i}}$$ (30)

These variables have values within the [0, 1] range, and the sum of their values equals 1. Using Eq (30), the probability of selecting the best solution is computed by simulating a Roulette wheel, where the optimal solutions from each group are considered. If the α-numeric parameter is close to value 1, and the β-numeric parameter is close to value 0, the intensification will be increased. Also, if the β-numeric parameter is close to value 1, and the α-numeric parameter is close to value 0, it leads to increasing diversity.

### 5.4. Second phase: Computing vultures starvation rate

Vultures frequently seek food, and if they are satiated, they have greater stamina and endurance to travel longer distances in search of food. When they are hungry, they lack the stamina and endurance needed to fly long distances and become aggressive. This behaviour helps to shift from the exploration to the exploitation phase, based on the vulture’s starvation rate as shown in Eq (33) and it is mathematically modeled by using Eq (31).


$$V_{sr(w)}^{i}=(2\;x\;{rand}_{w1}^{i}+1)x\;Z^{i}\;x(1-\frac{{itr}^{i}}{{mitr}^{i}})+t^{i}$$ (31)

$$t^{i}=h^{t}\;x({sin}^{k}(\frac{\pi }{2}\;x\;\frac{{itr}^{i}}{{mitr}^{i}})+cos(\frac{\pi }{2}\;x\;\frac{{itr}^{i}}{{mitr}^{i}})-1)$$ (32)

Where **V**_**sr**_ denotes that the W^th^ vultures’ starvation rate at the i^th^ iteration. ${rand}_{w1}^{i}$ is a random number between [0, 1], **Z**^**i**^ is a random number between [–1, 1] that changes at each iteration, the value gives the vultures hunger state based on the condition given in Eq (34). **itr**^**i**^ denotes the current iteration number, **mitr**^**i**^ denote the total number of iterations, k is a parameter with a fixed number set which indicates the optimization operation based on the condition given in Eq (35). **h**^**t**^ is a random number between [− 2, 2]. **t**^**i**^ is calculated by using Eq (32).


$$V_{sr}=\left\{ \begin{array}{c} if|V_{sr}|>1\;then\;AVOCA\;enters\;exploration\;phase\{\;searching\;for\;new\;food\}\\ if|V_{sr}|<1,\;then\;AVOCA\;enters\;exploitation\;phase\{\begin{array}{c} \;searching\;for\;\\ food\;in\;neighborhood\; \end{array}\} \end{array}\right.$$ (33)

$$Z^{i}=\left\{ \begin{array}{c} if\;Z^{i}<0,\;then\;the\;vulture\;is\;starved\\ if\;Z^{i}>0,\;then\;the\;vulture\;is\;satiated \end{array}\right.$$ (34)

$$K=\left\{ \begin{array}{c} f\;K\;{↑}_{ces}\;vulture\;entering\;the\;exploration\;phase\;gets\;{↑}_{ces}\;\\ if\;K\;{↓}_{ces}\;vulture\;entering\;the\;exploration\;phase\;gets\;{↓}_{ces} \end{array}\right.$$ (35)

When tackling optimization challenges, by the end of the exploration phase there’s no assurance that the final dataset will contain accurate solutions. This often leads to premature convergence in local optima. Eq (31) has been incorporated for solving complex optimization problems and escaping from local optima which in return enhances search space for the global optimum.

### 5.5. Third phase: Exploration stage

When |V_sr_|>1, vultures enter into the exploration phase and it is mathematically represented by using Eq (36). Finding food in the natural environment will be very difficult, so vultures search for new food at different locations through two different tactics based on the condition. To select any of the strategies, a random number between 0 and 1 is generated. If $P_{1}\geq {rand}_{p1}^{i}$ condition is satisfied then Eq (37) is used to calculate the Er_1_ and if $P_{1}<{rand}_{p1}^{i}$ is satisfied then Eq (40) is used to calculate the Er_2_.


$$P_{w}^{i+1}=\left\{ \begin{array}{l} {Er}_{1}\;if\;P_{1}\geq {rand}_{p1}^{i}\;then\;Elite-guiding\;strategy\;\\ {Er}_{2}\;if\;P_{1}<{rand}_{p1}^{i}\;then\;Random-search\;strategy \end{array}\right.$$ (36)

$${Er}_{1}=R_{w}^{i}-D_{w}^{i}\;x\;V_{sr(w)}^{i}$$ (37)

$$D_{w}^{i}=|W\;x\;R_{w}^{i}-P_{w}^{i}|$$ (38)

W=2×rand (39)

$${Er}_{2}=R_{w}^{i}-V_{sr(w)}^{i}+{rand}_{w2}^{i}\;x((U_{b}-L_{b})x{rand}_{w3}^{i}+L_{b})$$ (40)

Where $P_{w}^{i+1}$ is the w^th^ vultures’ position at i+1^th^ iteration, ${rand}_{p1}^{i},\;{rand}_{w2}^{i}$, are random numbers that follow uniform distribution in the range [0,1], $R_{w}^{i}$ represents the strongest social leader which is selected based on Eq (29). $D_{w}^{i}$ is the distance that exists between the previous best vulture and the current optimal vulture, and it can be calculated by using Eq (38). $V_{sr(w)}^{i}$ represents the vulture starvation rate which can be calculated by using Eq (31). Vultures move in random motion to protect food from other vultures which is represented by the coefficient vector W and can be calculated using Eq (39), where rand is a random number between [0,1] and it changes with each iteration. $P_{w}^{i}$ is the current vector position of the vulture. ${rand}_{w3}^{i}$ is used to increase the coefficient of random nature coefficient. If it takes a number close to 1, then it distributes the solutions with similar patterns. It also creates a high random coefficient at the search environment scale to increase diversity and search for different search space areas.

### 5.6. Fourth phase: Exploitation stage

When |V_sr_|<1, vultures enter into the exploitation phase and this is further subdivided into two additional phases, each governed by specific strategies and controlled by the parameters P2 and P3, as outlined in Eq (41). P2 and P3 are used to choose strategies available in the first and second phases. These parameters should fall within the [0,1] range and must be performed before the search operation.


$$V_{sr}=\left\{ \;\begin{array}{c} if|V_{sr}|>0.5,\;enters\;P_{2}\;phase\;\\ if|V_{sr}|\leq 0.5,\;enters\;P_{3}\;phase\; \end{array}\right.$$ (41)

#### 5.6.1. Exploitation (1st phase)

When the value |V_sr_| falls within the range of [0.5 1], the AVOCA enters the first phase of Exploitation. In the first phase, two different strategies are carried out as shown in Eq (42) based on the generated P2 and ${rand}_{p2}^{i}$ the value which lies between [0 1]. $if\;P_{2}\geq {rand}_{p2}^{i}$ condition is satisfied then Et_1_ strategy is selected and calculated by using Eq (43) and $if\;P_{2}<{rand}_{p2}^{i}$ condition is satisfied then Et_2_ strategy is selected and calculated by using Eq (45). When |V_sr_| is ≥ 0.5, which signifies that vultures have enough energy. However, the congregation of many vultures around a single food source can give rise to significant conflicts during food acquisition. In such situations, physically powerful vultures opt not to share food with other vultures. In contrast, the weaker vultures try to tire and steal food from the strongest vultures by gathering around them and engaging in minor conflicts and it is represented by using Eq (43). Vultures frequently use rotational flight strategy to model Spiral Motion which is represented by using Eq (45).


$$P_{w}^{i+1}=\left\{ \begin{array}{c} {Et}_{1}\;if\;P_{2}\geq {rand}_{p2}^{i}\;then\;Siege-fight\;strategy\;\\ {Et}_{2}\;if\;P_{2}<{rand}_{p2}^{i}\;then\;Rotating-flight\;strategy \end{array}\right.$$ (42)

$${Et}_{1}=D_{W}^{i\;}\;x(V_{sr(w)}^{i}+{rand}_{w4}^{i})-d_{w}^{i}$$ (43)

$$d_{w}^{i}=R_{w}^{i}-P_{w}^{i}$$ (44)

$${Et}_{2}=R_{W}^{i}-(S_{w1}^{i}+S_{w2}^{i})$$ (45)

$$S_{w1=}^{i}R_{w}^{i}\;x(\frac{{rand}_{w5}^{i}XP_{w}^{i}}{2\pi })x\;cos(P_{w}^{i})$$ (46)

$$S_{w2=}^{i}R_{w}^{i}\;x(\frac{{rand}_{w6}^{i}XP_{w}^{i}}{2\pi })x\;sin(P_{w}^{i})$$ (47)

Where $D_{w}^{i}$ represents the distance between the previous best vulture and the current optimal vulture, $V_{sr(w)}^{i}$ represents the vulture starvation rate of vultures, ${rand}_{w4}^{i},\;{rand}_{w5}^{i}$, and ${rand}_{w6}^{i}$ are the random number that lies between [0,1], $S_{w1}^{i}$ and $S_{w2}^{i}$ represent the two best vultures by using spiral motion which can be calculated by using Eq (46) and Eq (47).

#### 5.6.2. Exploitation:(2nd phase)

When the value |V_sr_| < 0.5, the AVOCA progresses into the second phase of Exploitation. Initially, the majority of vultures in the population appear satiated. However, after some time, the two strongest vultures display signs of hunger and feeble. During this phase, vultures become aggressive in their pursuit of food represented by using Eq (52), and several vultures will congregate around a specific food source represented by Eq (49). Based on the condition given in Eq (48) the fighting strategy is selected. If Et_3_ is selected then it is calculated by using Eq (49) or else if Et_4_ is selected then it is calculated by using Eq (52).


$$P_{w}^{i+1}=\left\{ \begin{array}{c} {Et}_{3}\;if\;P_{3}\geq {rand}_{p3}^{i}\;\\ {Et}_{4}\;if\;P_{3}<{rand}_{p3}^{i}\; \end{array}\right.$$ (48)

$${Et}_{3}=\frac{A_{w1}^{i}+A_{w2}^{i}}{2}$$ (49)

$$A_{w1}^{i}={BV}_{w1}^{i}-\frac{{BV}_{w1}^{i}\;x\;P_{w}^{i}\;}{{BV}_{w1}^{i}-{\left(P_{w}^{i}\right)}^{2}}\;x\;V_{sr(w)}^{i}$$ (50)

$$A_{w2}^{i}={BV}_{w2}^{i}-\frac{{BV}_{w2}^{i}\;x\;P_{w}^{i}\;}{{BV}_{w2}^{i}-{\left(P_{w}^{i}\right)}^{2}}\;x\;V_{sr(w)}^{i}$$ (51)

$${Et}_{4}=R_{w}^{i}-|d_{w}^{i}|x\;V_{sr(w)}^{i}x\;levy(d)$$ (52)

$$levy(dim)=0.01\;x\;\frac{u\;x\;\sigma }{{\left|v\right|}^{\frac{1}{\beta }}}$$ (53)

$$\sigma ={\left(\frac{\Gamma (1+\beta \;x\sin\left(\frac{\pi \beta }{2}\right)}{\Gamma (1+\beta_{2})x\;\beta \;x\;2(\frac{\beta -1}{2})}\right)}^{\frac{1}{\beta }}$$ (54)

**Fig 3:**
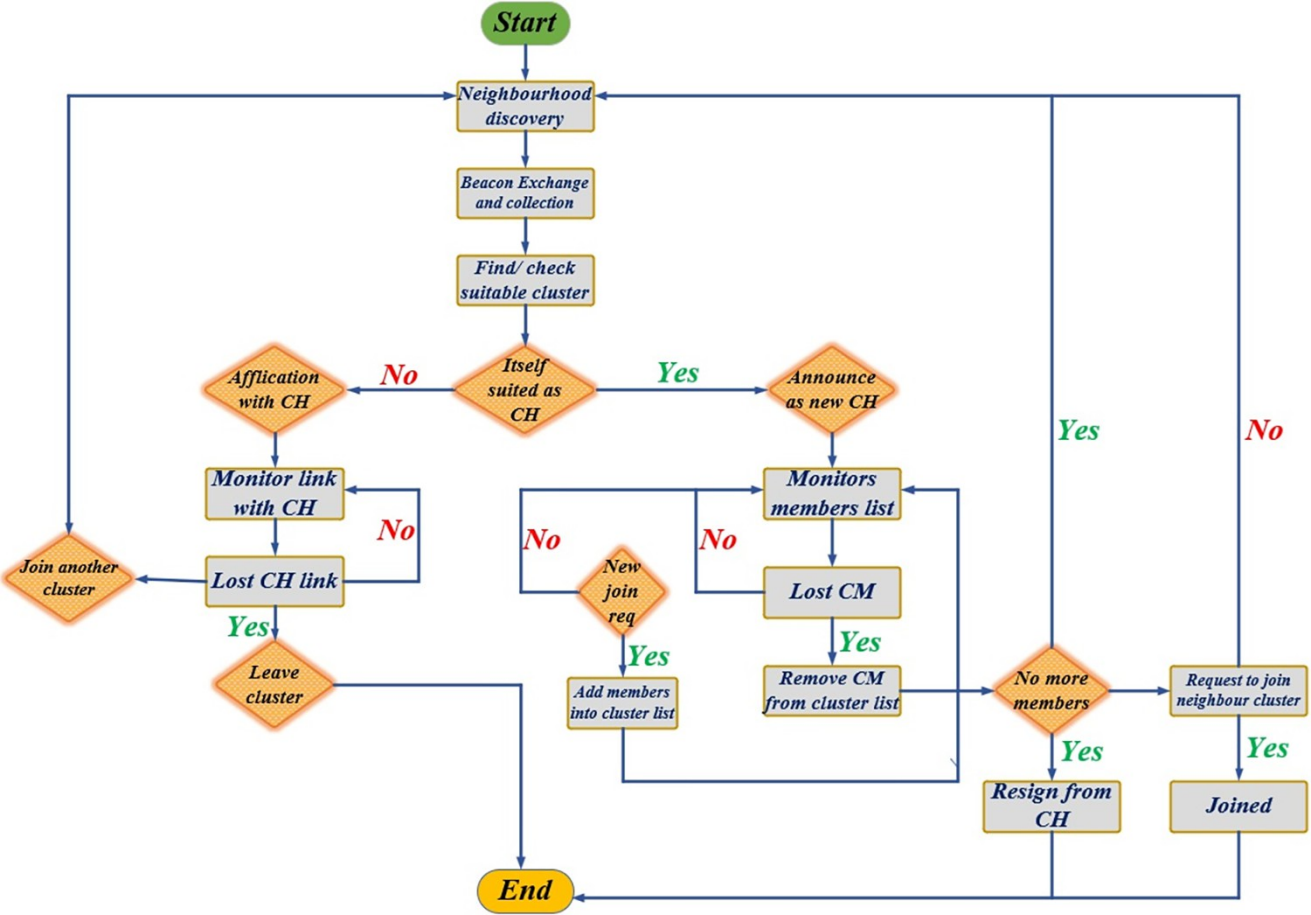
General procedural flow chart for AVOCA.

Where $A_{w1}^{i}$ and $A_{w2}^{i}$ represent the vultures congregate around a specific food source and the values of $A_{w1}^{i}$ and $A_{w2}^{i}$ is calculated by using Eq (50) and Eq (51), ${BV}_{w1}^{i}\&{BV}_{w2}^{i}$ is the strongest vulture of the first and second group in the current iteration, $P_{w}^{i}$ indicates the current vector position of the vulture, $V_{sr(w)}^{i}$ represents the vulture starvation rate of vultures, levy(d) represent the levy fight which is used to increase the effectiveness of the algorithm which is calculated by using (53), u & v are a random number that ranges between [0,1], and β is a fixed and default number is 1.5. and σ is calculated by using Eq (54). A flowchart illustrating these phases is provided in Fig 3 for better understanding and the pseudocode for the AVOCA is illustrated in Table 10.

**Table 10:** AVOCA approach.

**Algorithm 1:** Pseudocode for the proposed AVOCA
1. **Input:** The Vultures population Wi (i = 1, 2. . ., n)
2. Initialize each vehicle’s position, direction, and speed.
3. Establish a mesh topology with nodes/vertices, as each vertex tries to represent the vehicle id.
4. **While** (*itr*^*i*^<*mitr*^*i*^) **do**
5. Calculate the fitness in each iteration
6. Return BV_1_ and BV_2_ as FSLV and SSLV
**7. For** (each Vulture (P_*i*_)) **do**
8. Select R(i) using Eq (29)
9. **If** vulture is satiated,
10. High energy, strength to fly longer distances in search of food.
11. **Else**
12. Can’t fly due to low energy, calculate vulture starvation rate using Eq (31)
13. **End If**
14. **End For**
15. **If |***V*_*sr*_**|**>1 (Enters exploration phase)
16. **If** ($P_{1}\geq {rand}_{p1}^{i})$ then
17. Update the location Vulture using Eq (37)
18. **Else**
19. Update the location Vulture using Eq (40)
20. **End If**
21. **End If**
22. **If** ((**|***V*_*sr*_**|**<1) **&** (|*V*_*sr*_| ≥ 0.5)) (Enters Exploitation phase)
23. **If** $P_{2}\geq {rand}_{p2}^{i}$ then
24. Update the location Vulture using Eq (43)
25. **Else**
26. Update the location Vulture using Eq (45)
27. **End If**
28. **Else**
29. **If** ((|*V*_*sr*_|<1) **&** (|*V*_*sr*_| < 0.5))
30. **If** $P_{3}\geq {rand}_{p3}^{i}$ then
31. Update the location Vulture using Eq (49)
32. **Else**
33. Update the location Vulture using Eq (52)
34. **End If**
35. **End If**
36. **End If**
37. **End While**
38. **Output:** Return the most optimal solution found in the search space

### 5.7. Equilibrium phase

The equilibrium should be maintained between the exploration and exploitation phases to prevent premature convergence in the exploitation phase and to maintain diversity in the exploration phase and it is calculated by using Eq (55) concerning cluster generation. If **w**_**explore**_ is higher, the optimization algorithm will prioritize exploration, which determines the formation of new clusters by using Eq (56). **w**_**explore**_ actively seeking diverse solutions across the solution space where the optimal solution is not well-defined. Conversely, if **w**_**exploit**_ is higher, it will prioritize exploitation, which determines the size of the known clusters by using Eq (57). **w**_**exploit**_ intensifying the search for known promising solutions. It is advantageous when the algorithm aims to refine optimized solutions in the vicinity of known optima and quick convergence is essential.


$$f(x)=w_{explore}.Explore(x)+w_{exploit}.Exploit(x)$$ (55)

$$Explore(x)=\frac{1}{N}\sum_{i=1}^{N}{clusters}_{new}(x_{i})$$ (56)

$$Exploit(x)=\frac{1}{N}\sum_{i=1}^{N}{size}_{known}(x_{i})$$ (57)

Where f(x) is the overall objective function representing the equilibrium between exploration and exploitation, **w**_**explore**_ and **w**_**exploit**_ are the weights assigned to that component, N is the number of vehicles, and **clusters**_**new**_(**x**_**i**_) is the number of new clusters formed by vehicle I and **size**_**known**_(**x**_**i**_) is the size of the known cluster utilized by vehicle i.

### 5.8. Computational complexity

The computational complexity of the AVOCA is determined by three imperative processes: initialization, fitness evaluation, and vulture updating. The computational complexity of the initialization phase is equivalent to O(N) for N vultures. Furthermore, the computational complexity of the update mechanism process, which involves searching for the optimal location and updating the location vector, is equivalent to O (T **x** N) + O (T **x** N **x** D). As a result of the above explanation, the computational complexity of the AVOCA is equivalent to O (N **x** (T **+** TD)). Where N represents no. of vultures, T is the maximum number of iterations, and D is the problem dimension. The computational complexity of AVOCA is compared with the state-of-art-algorithms is illustrated in Table 11.

**Table 11:** Computational complexity comparison with state-of-art-algorithms.

	Time Complexity	Space Complexity	Computational Complexity
CAMONET	*O*(*tn*^2^)	*O*(*nd*+*d*^2^*logN*)	*O*(*tn*^2^+*tnd*)
SAMNET	*O*(*TJ*^2^+*J*^4^)	*O*(*J*^2^)	O (*J*^4^)
I-WOA	*O*(N*D+f)	*O*(N*D)	*O*(*ND*)
HHOCNET	*O*(*N*)	*O*(*T*X*N*)+*O*(*T*X*N*X*D*)	*O*(*N*X(*T*+*TD*+1)
AVOCA	*O*(*N*)	*O*(*T*X*N*)+*O*(*T*X*N*X*D*)	*O*(*N*X(*T*+*TD*)

## 6. Implementation and results analysis for AVOCA

The experiments were conducted with an AMD Ryzen 3 processor with 2.60 GHz clock speed and 8 GB of RAM, using MATLAB version R2020a for a highway scenario. The nodes move bidirectionally, with a node count ranging between 30 and 60. Four different sizes of road segments were used, varying from 1 X 1km^2^ to 4 X 4km^2^ grid size. The degree difference value in the ad hoc network is set to 10 for load balancing. The experiment is conducted by using the IEEE 802.11p MAC protocol. In addition to AVOCA, this research also implemented several well-renowned algorithms for clustering in VANET, including CAMONET [29], SAMNET [37], I-WOA [21], and HHOCNET [36]. As shown in Table 12, all the algorithms were set to have analogous parameter values.

**Table 12:** Simulation parameters set analogously for all of the algorithms.

Parameters	CAMONET	SAMNET	I-WOA	HHOCNET	AVOCA
Particle population size	100	100	100	100	100
Maximum no. of iterations	150	150	150	150	150
Weight of inertia W	0.694	0.694	0.694	0.694	0.694
$C_{1}^{1}$	2	2	2	2	2
$C_{2}^{1}$	2	2	2	2	2
The range of vehicle velocity (meter /s)	22–30	22–30	22–30	22–30	22–30
Maximum acceleration (meter /s2)	1.5	1.5	1.5	1.5	1.5
Simulation area (Km X KM)	1 × 1 to 4 × 4	1 × 1 to 4 × 4	1 × 1 to 4 × 4	1 × 1 to 4 × 4	1 × 1 to 4 × 4
Minimum V2V distance (meters)	2	2	2	2	2
Maximum V2V distance (meters)	5	5	5	5	5
Lane width (meters)	50	50	50	50	50
Transmission range (meters)	100–600	100–600	100–600	100–600	100–600
Mobility model	Freeway	Freeway	Freeway	Freeway	Freeway
No. of simulations	10	10	10	10	10
Nodes	30,40,50,60	30,40,50,60	30,40,50,60	30,40,50,60	30,40,50,60
W1 (weight of first objective function)	0.5	0.5	0.5	0.5	0.5
W2 (weight of second objective function)	0.5	0.5	0.5	0.5	0.5
Total no. of lanes	8	8	8	8	8

### 6.1. Transmission ranges vs no. of clusters

Several factors influence the number of clusters, including a node’s transmission range and grid size. According to the analysis, the transmission range and the no. of clusters generated exhibit an inversely proportional relationship represented by Eq (58). When the transmission range is minimized, a greater no. of clusters is formed due to reduced connectivity but when the transmission range gradually increases, the number of clusters generated decreases due to larger area connectivity which results in more isolated groups.


$${VN}_{c}\alpha \frac{1}{R^{2}}$$ (58)

Where R represents the transmission range and ***VN***_***c***_ represents the number of clusters generated.

#### 6.1.1. For 1km x 1km grid size

**Fig 4:**
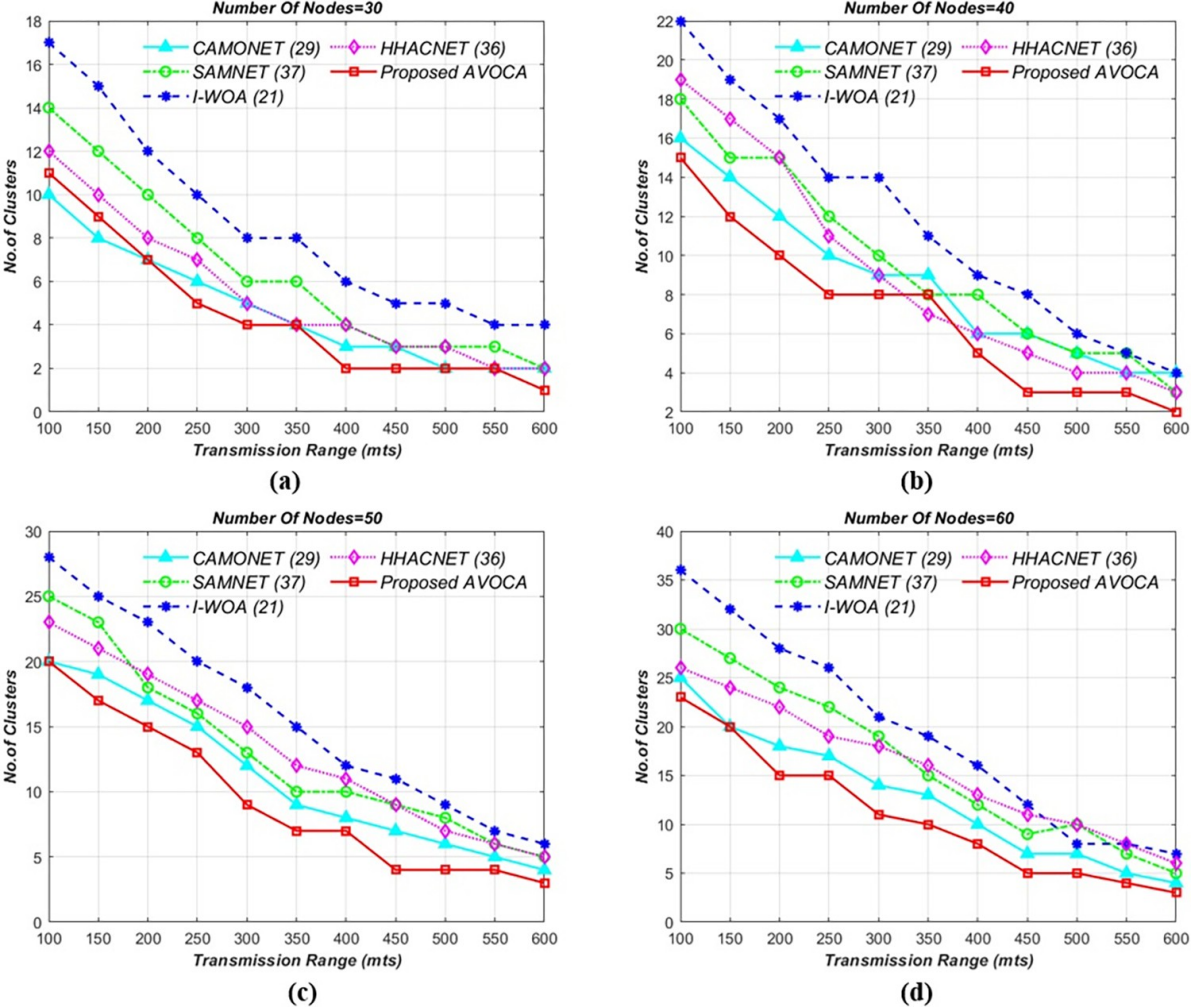
Tx range vs. no. of clusters for different node counts i.e. (a) 30 (b) 40 (c) 50, and (d) 60 for 1km X 1km grid.

In the initial experiment, the road segment size is kept constant at 1 km x 1 km, and the transmission range is varied from 100 to 600 meters, with node counts ranging from 30 to 60. The proposed AVOCA algorithm identifies the best solutions by exploring the search space and the experimental results are graphically depicted in Fig 4. By varying the transmission range for different node counts i.e 30, 40, 50, and 60 the proposed AVOCA algorithm generates a maximum of 11, 15, 20, and 23 clusters for minimum transmission range but generates optimal no.of clusters for the maximum transmission range as shown in Fig 4(A)-4(D). The proposed AVOCA algorithm generates 30% lesser no. of clusters when compared with CAMONET, 59% lesser no. of clusters when compared with SAMNET, 70% lesser no. of clusters when compared with I-WOA, and 52% lesser no. of clusters when compared with HHOCNET. The graphs in Fig 4 demonstrate that the proposed AVOCA algorithm consistently produces optimal results in terms of clusters when compared to the CAMONET, SAMNET, I-WOA, and HHOCNET.

#### 6.1.2. For 2km x 2km grid size

**Fig 5:**
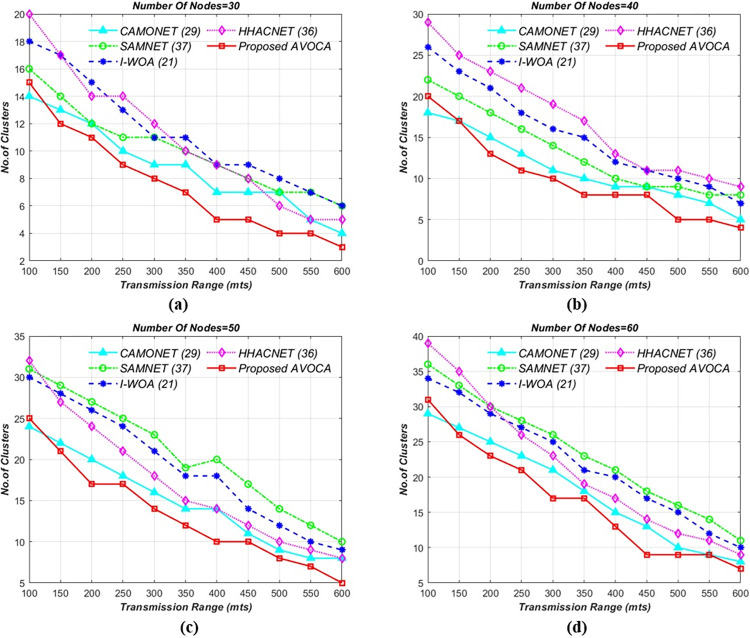
Tx range vs. no. of clusters for different node counts i.e. (a) 30 (b) 40 (c) 50, and (d) 60 for 2km X 2km grid.

The road segment size was adjusted to a 2 km x 2 km grid and the other remaining parameters remained unchanged as initial simulations. The results of this simulation are visually represented in Fig 5, which summarizes the inter-correlation of the four approaches. For the different node counts i.e 30, 40, 50, and 60 the proposed AVOCA algorithm generates a maximum of 15, 20, 25, and 31 clusters for minimum transmission range but generates optimal clusters i.e 3, 4, 5, and 7 for the maximum transmission range as shown in Fig 5(A)–5(D). The proposed AVOCA algorithm generates 20% lesser no. of clusters when compared with CAMONET, 56% lesser no. of clusters when compared with SAMNET, 58% lesser no. of clusters when compared with I-WOA, and 53% lesser no. of clusters when compared with HHOCNET.

#### 6.1.3. For 3 x 3Km^2^ and 4 x 4Km^2^ grid size

**Fig 6:**
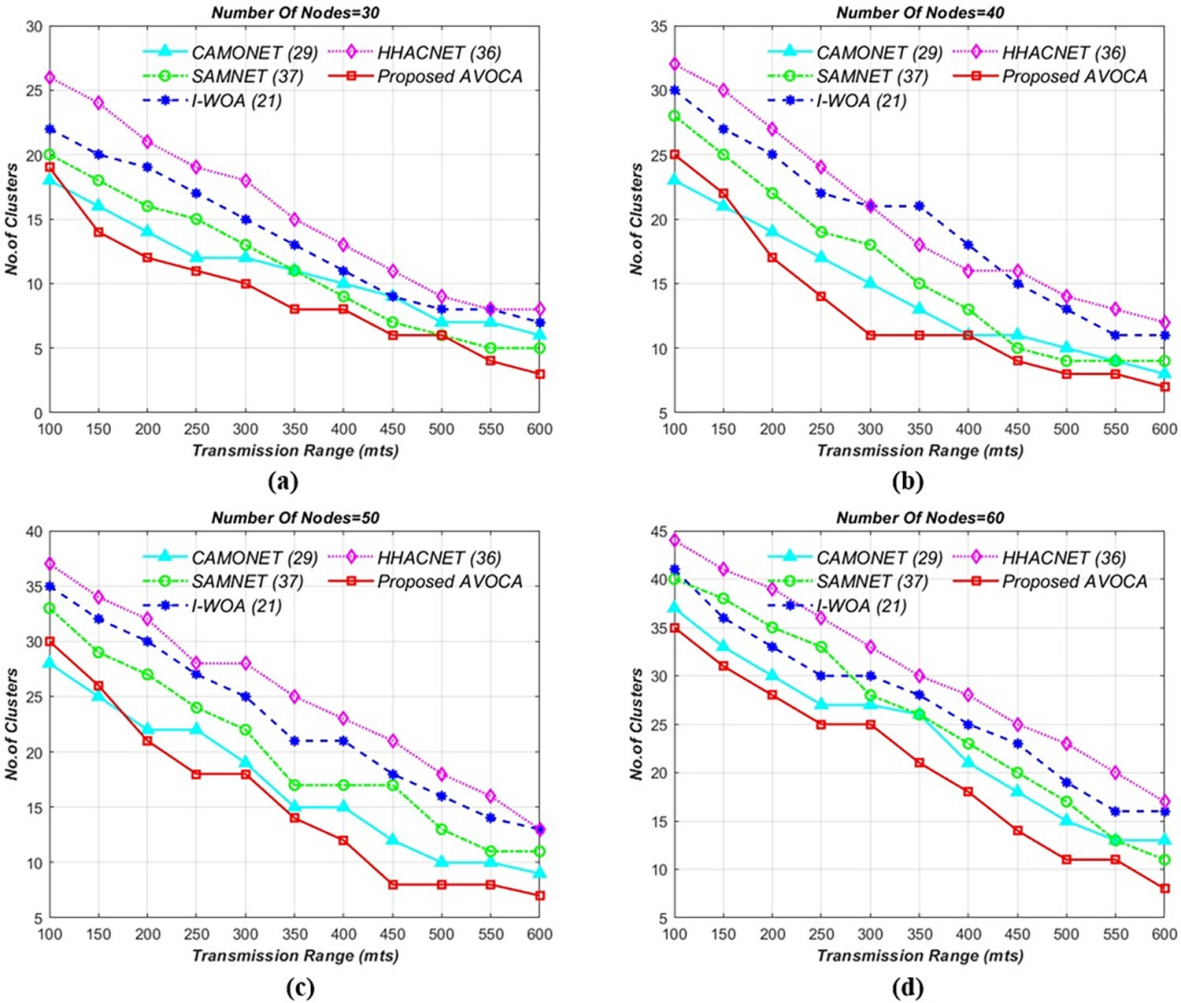
Tx range vs. no. of clusters for different node counts i.e. (a) 30 (b) 40 (c) 50, and (d) 60 for 3km X 3km grid.

**Fig 7:**
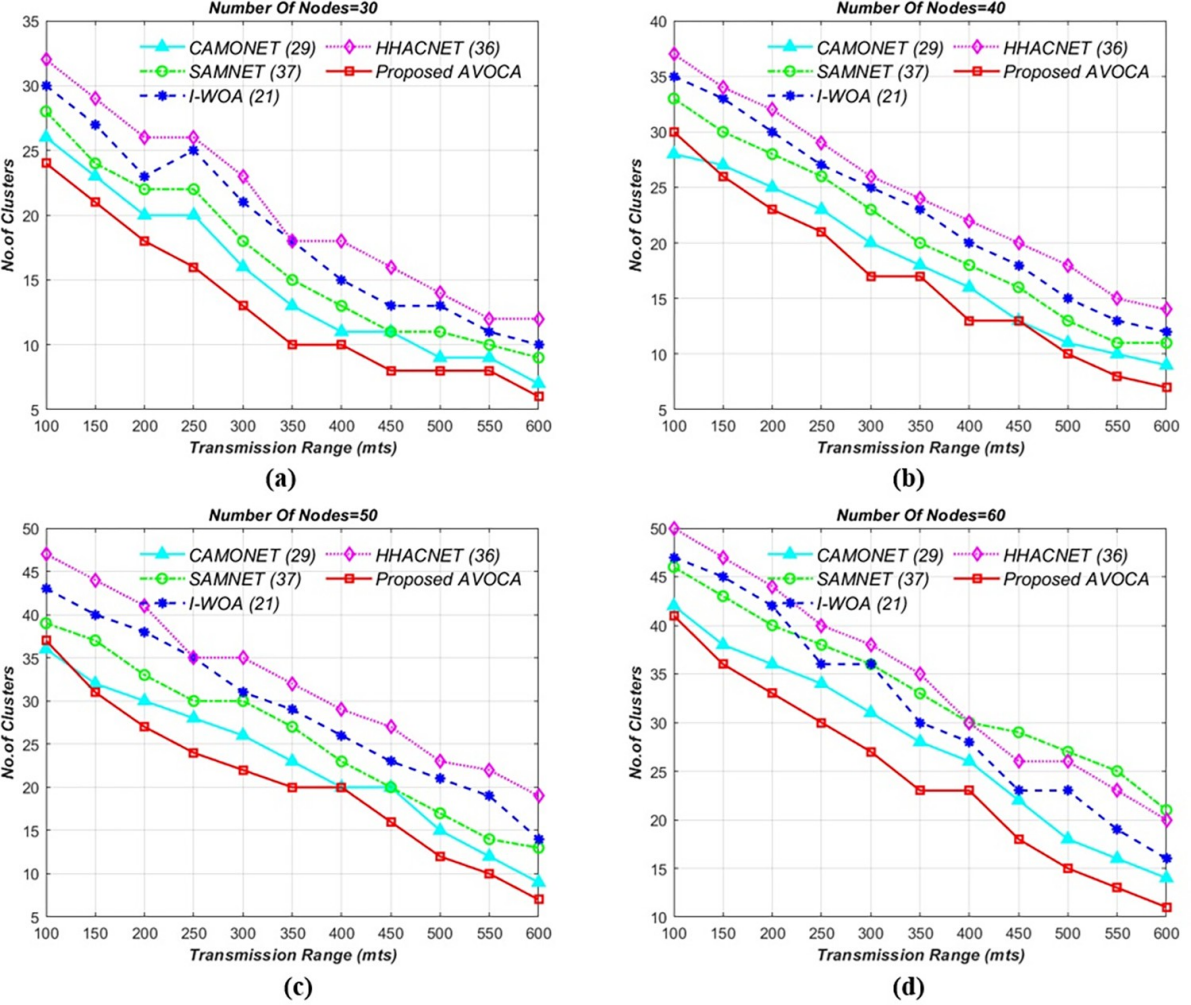
Tx range vs. no. of clusters for different node counts i.e. (a) 30 (b) 40 (c) 50, and (d) 60 for 4kmX 4km grid.

The setup experiment is not changed but the grid size is gradually increased. The results for 3**x**3km^2^ grid size are schematically represented as shown in Fig 6 which illustrates that AVOCA still performs better than other algorithms. For the different node counts i.e 30, 40, 50, and 60 the proposed AVOCA algorithm generates a maximum of 19, 25, 30, and 35 clusters for minimum transmission range but generates optimal clusters i.e 3, 7, 7, and 8 for the maximum transmission range as shown in Fig 6(A)–6(D). The proposed AVOCA algorithm generates 22% lesser no. of clusters when compared with CAMONET, 32% lesser no. of clusters when compared with SAMNET, 59% lesser no. of clusters when compared with I-WOA, and 77% lesser no. of clusters when compared with HHOCNET. The results for 4**x**4km^2^ grid size are schematically represented as shown in Fig 7. For the different node counts i.e 30, 40, 50, and 60 the proposed AVOCA algorithm generates a maximum of 24, 30, 37, and 41 clusters for minimum transmission range but generates optimal clusters i.e 6, 7, 7, and 11 for the maximum transmission range as shown in Fig 7(A)–7(D). The proposed AVOCA algorithm generates 16% lesser no. of clusters when compared with CAMONET, 38% lesser no. of clusters when compared with SAMNET, 48% lesser no. of clusters when compared with I-WOA, and 66% lesser no. of clusters when compared with HHOCNET.

Due to the algorithms’ unpredictable behavior, the AVOCA results occasionally overlap with other methods because bio-inspired strategies are randomly initialized or even fine-tuned using a probabilistic method, and intelligent self-adaptation weights are assigned in the next iteration.

### 6.2. Network nodes vs no. of clusters

In this scenario, the experiment is conducted by varying network nodes from 30 to 80 for different transmission ranges i.e., 100m, 200m, 300m, and 400m for different grid sizes i.e., (1 x 1 km^2^ to 4 x 4 km^2^). To ensure consistency, we keep the transmission range constant as the number of network nodes increases. The results are compared with those of state-of-the-art competitors.

#### 6.2.1. For 1 x 1Km^2^ grid size

**Fig 8:**
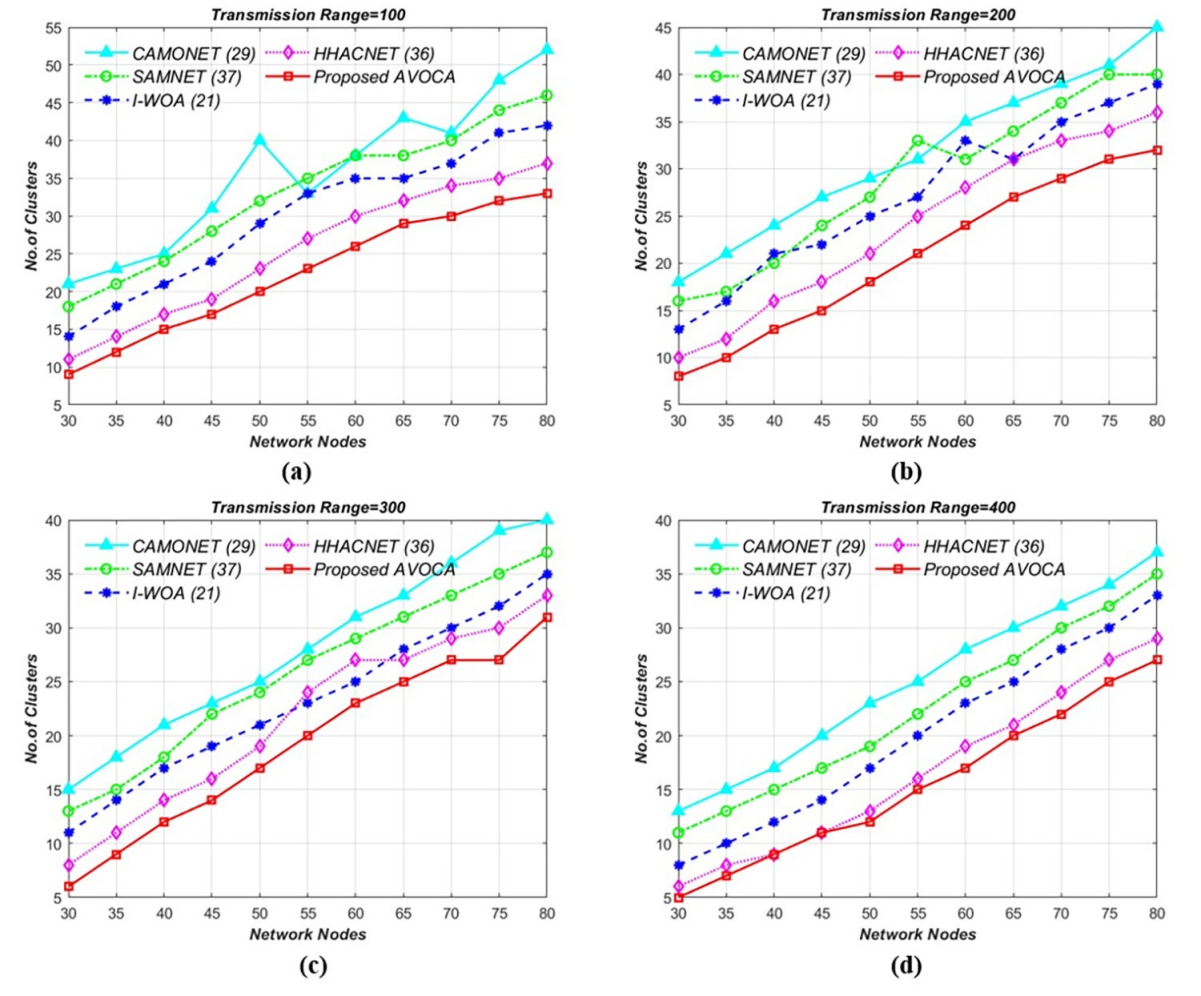
Network nodes vs. no. of clusters for different Tx Range (mts) i.e. (a) 100 (b) 200 (c) 300, and (d) 400 for 1 **x** 1 km^2^ Grid Size.

In the initial scenario, the road segment size is kept constant at 1 km x 1 km, and the transmission range is from 100 to 400 meters, with node counts varying from 30 to 60. Fig 8 illustrates the schematic relation between the number of clusters generated while varying network nodes. By varying the network nodes from 30 to 80 for different transmission ranges i.e 100, 200, 300, and 400 mts the proposed AVOCA algorithm generates 9, 8, 6, and 5 clusters for 30 nodes and generates 33, 32, 31, and 27 clusters when network nodes are increased gradually to 80 nodes as shown in Fig 8(A)–8(D). The proposed AVOCA algorithm generates 73% lesser no. of clusters when compared with CAMONET, 55% lesser no. of clusters when compared with SAMNET, 37% lesser no. of clusters when compared with I-WOA, and 15% lesser no. of clusters when compared with HHOCNET. Our proposed AVOCA algorithm requires fewer clusters to cover the entire network compared to other state-of-the-art algorithms.

#### 6.2.2. For 2 x 2Km^2^ grid size

**Fig 9:**
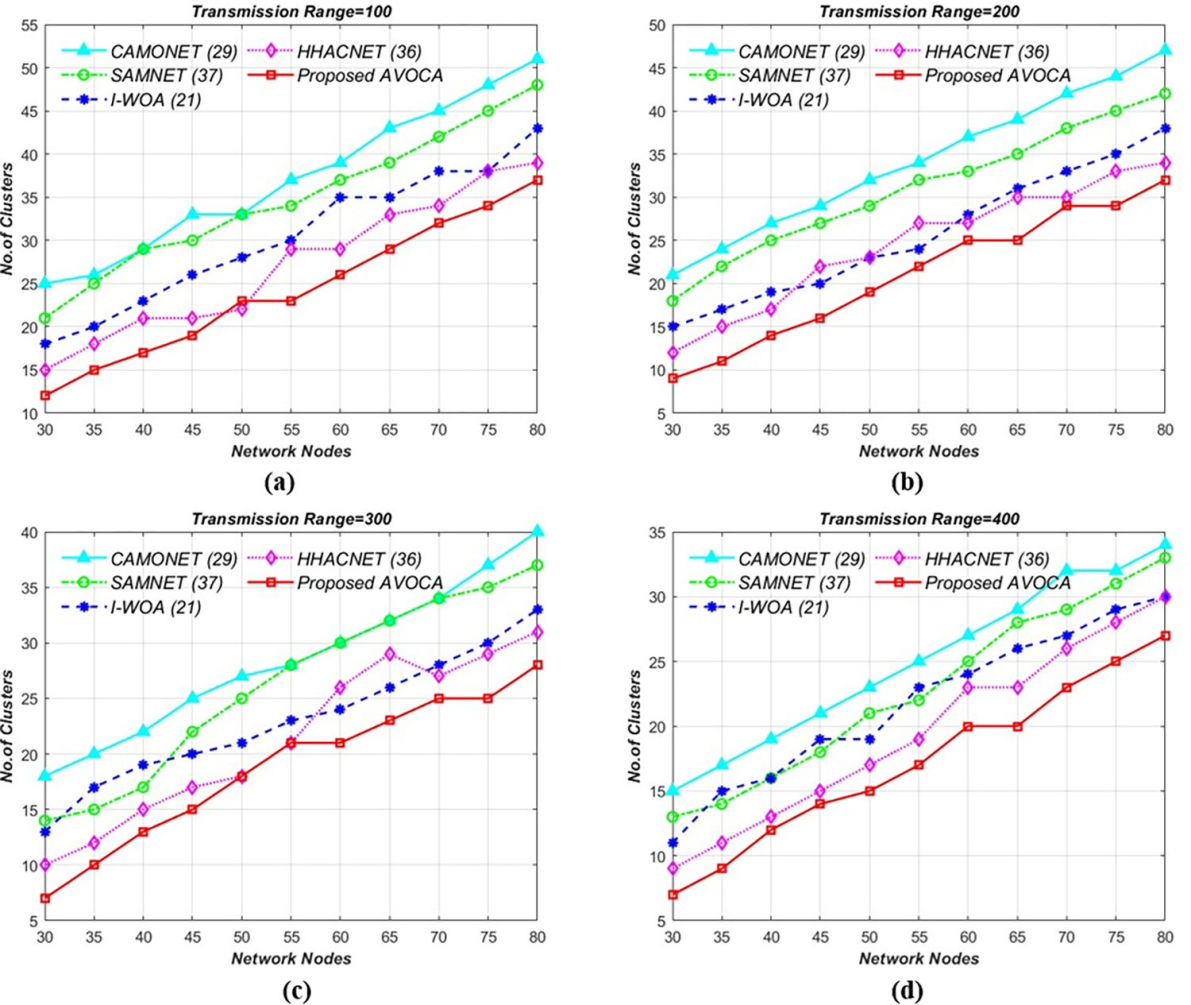
Network nodes vs. no. of clusters for different Tx Range (mts) i.e. (a) 100 (b) 200 (c) 300, and (d) 400 for 2 **x** 2Km^2^ Grid Size.

**Fig 10:**
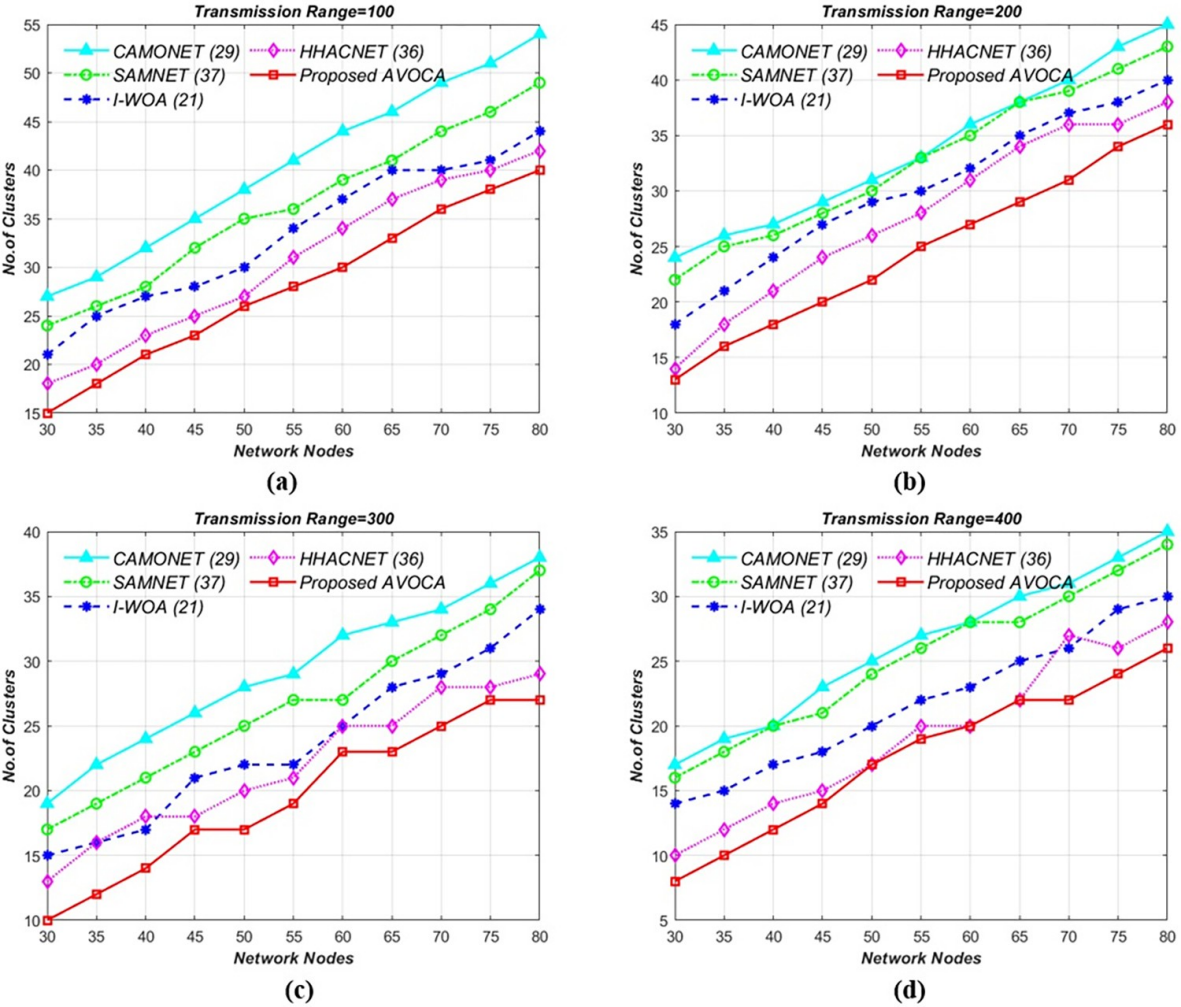
Network nodes vs. no. of clusters for different Tx Range (mts) i.e. (a) 100 (b) 200 (c) 300, and (d) 400 for 3 **x** 3Km^2^ Grid Size.

Following the initial simulations, the road segment size was adjusted to a 2 km x 2 km grid, while the remaining parameters remained unchanged. The results for the stated scenario are schematically represented as shown in Fig 9. By varying the network nodes from 30 to 80 for different transmission ranges i.e 100, 200, 300, and 400 mts the proposed AVOCA algorithm generates 12, 9, 7, and 7 clusters for 30 nodes and generates 37, 32, 28, and 27 clusters when network nodes are increased gradually to 80 nodes as shown in Fig 10(A)–10(D). The proposed AVOCA algorithm generates 43% lesser no. of clusters when compared with CAMONET, 30% lesser no. of clusters when compared with SAMNET, 20% lesser no. of clusters when compared with I-WOA, and 11% lesser no. of clusters when compared with HHOCNET.

#### 6.2.3. For 3 x 3Km^2^ and 4 x 4Km^2^ grid size

**Fig 11:**
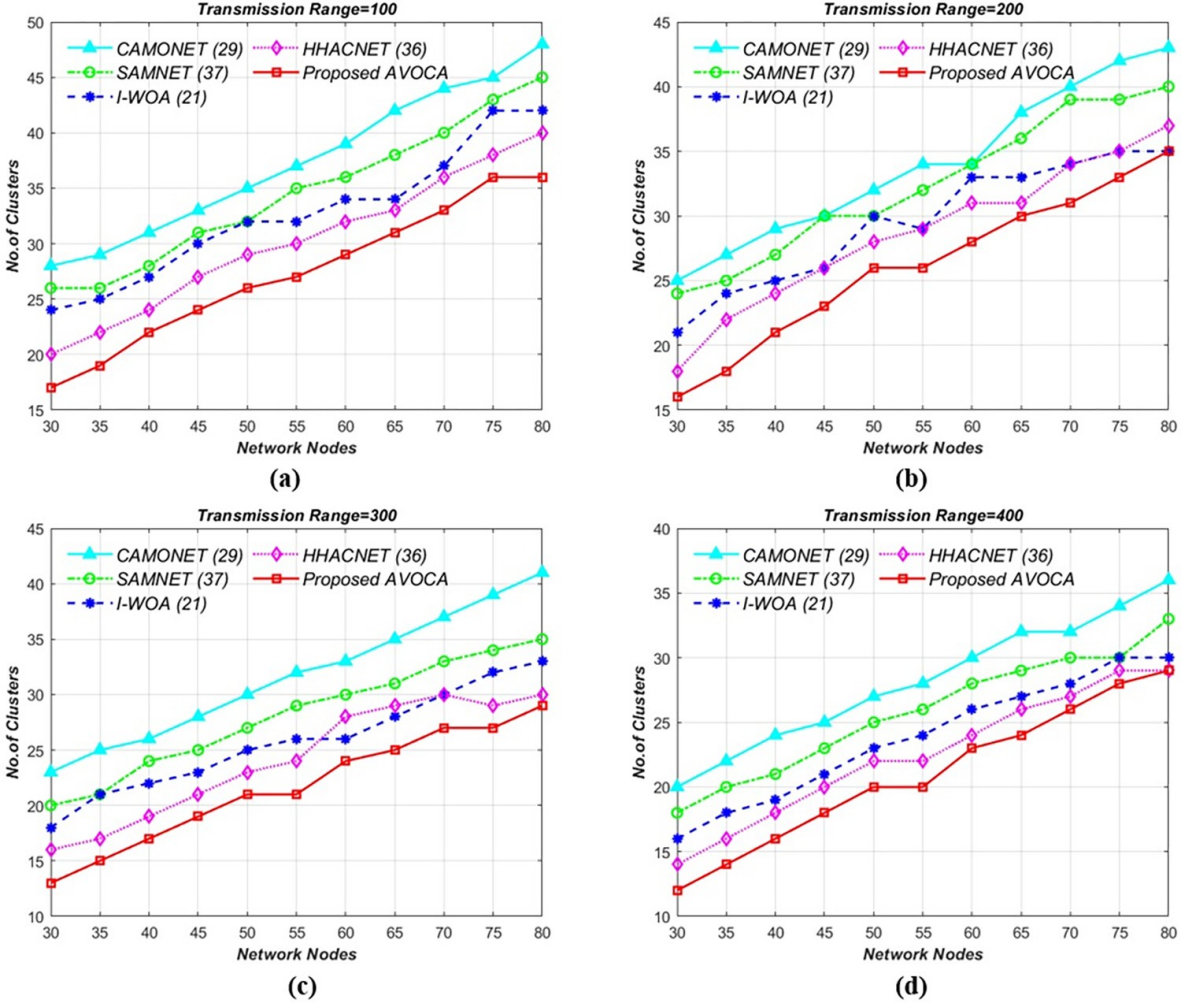
Network nodes vs. no. of clusters for different Tx Range (mts) i.e. (a) 100 (b) 200 (c) 300, and (d) 400 for 4 **x** 4Km^2^ Grid Size.

The setup experiment is not changed but the grid size is gradually increased. For 3**x**3km^2^ grid size, the results are schematically represented as shown in Fig 10, where the proposed AVOCA algorithm generates 15,13,10 and 8 clusters for 30 nodes and generates 40, 36, 27, and 26 clusters when network nodes are increased gradually to 80 nodes as shown in Fig 10(A)–10(D). The proposed AVOCA algorithm generates 55% lesser no. of clusters when compared with CAMONET, 43% lesser no. of clusters when compared with SAMNET, 27% lesser no. of clusters when compared with I-WOA, and 13% lesser no. of clusters when compared with HHOCNET. For 4**x**4km^2^ grid size, the results are schematically represented as shown in Fig 11, where the proposed AVOCA algorithm generates 17,16,13 and 12 clusters for 30 nodes and generates 36, 35, 29, and 29 clusters when network nodes are increased gradually to 80 nodes as shown in Fig 11(A)–11(D). The proposed AVOCA algorithm generates 55% lesser no. of clusters when compared with CAMONET, 43% lesser no. of clusters when compared with SAMNET, 27% lesser no. of clusters when compared with I-WOA, and 13% lesser no. of clusters when compared with HHOCNET.

According to analysis, the network nodes and the no. of clusters generated exhibit a direct proportional relationship represented by Eq (59). When the network nodes are minimum, optimal clusters are generated but when the network nodes are increased gradually the clusters generated also increase gradually.


$${VN}_{c}\alpha \;S_{nn}$$ (59)

Where **VN**_**c**_ represents the number of clusters generated and **S**_**nn**_ represent the size of the network nodes.

### 6.3. Network grid size vs cluster efficiency

Different network grid sizes which represent the spatial division of the VANET environment, significantly influence clustering efficiency and these are inversely proportional to each other as shown in Eq (60). Larger grid sizes tend to yield more vehicles to fall within a single grid cell, potentially leading to larger clusters. While this can enhance inter-vehicle communication within clusters, it may lead to increased communication overhead which indirectly reduces scalability and efficiency. Conversely, smaller grid sizes result in fewer vehicles within each grid cell, potentially leading to smaller, more dynamic clusters. This can contribute to improved efficiency by minimizing communication overhead and enhancing adaptability to changing network conditions. Selecting an optimal grid size is imperative for achieving an efficient balance between cluster formation, inter-cluster communication, and adaptability to the dynamic nature.


$$C_{eff}\alpha \frac{1}{Grid\;size}$$ (60)

## 7. Conclusion

VANETs endure dynamic properties that jeopardize scalability, routing, and, in some cases, security. These properties give rise to significant challenges such as stability, reliability, and QoS, which are NP-hard problems. The stability can be achieved by increasing the lifetime of a cluster, which is accomplished by generating optimal clusters. To achieve optimal clusters, an intelligent nature-inspired meta-heuristic African Vulture optimization-based Clustering Algorithm (AVOCA) is implemented as a vital effort in a VANET environment. The proposed algorithm can reduce network randomness and achieve network stability by effectively optimizing node clustering, taking into account parameters such as transmission range, node count, and grid size. Because of its evolutionary capability, the proposed AVOCA algorithm can process larger search spaces by dynamically adjusting self-adaptive weights. By varying transmission ranges and node count for different grid sizes ranging from 1 x 1 km^2^ to 4 x 4 km, AVOCA generates 40% less clusters when compared to the Clustering Algorithm Based on Moth-Flame Optimization for VANETs (CAMONET). AVOCA generates 45% less clusters when compared to Self-Adaptive Multi-Kernel Clustering for Urban VANETs (SAMNET), AVOCA generates 43% less clusters when compared to Intelligent Whale Optimization Algorithm (i-WOA) and AVOCA generates 38% less clusters when compared to Harris Hawks Optimization (HHO). The results show that AVOCA outperforms state-of-the-art algorithms in generating optimal clusters and the results are schematically represented. The generated results reveal that grid size and network nodes are directly proportional to the no. of clusters and the transmission range is inversely proportional to the no. of clusters. The proposed approach generates the optimal number of clusters with minimum cost and achieves stability, load optimization, and improved network utilization to ensure communication efficiency. However, several other factors like routing protocols, security, and signal interference can affect the stability which can be further incorporated into the proposed algorithm or by using other bio-inspired algorithms. The proposed algorithm can be executed in the live scenario to improve it further.

## Abbreviations

Abbreviation: Description
IoT: Internet of Things
IoV: Internet of Vehicles
IoE: Internet of Energy
ITS: Intelligent Transportation System
AU: Application Unit
OBU: On-board Unit
RSU: Road Side Unit
E2E: End-to-End
MOP: Multi-Objective Problems
EA: Evolutionary Algorithms
QoS: Quality of Service
P2P: Peer-to-Peer
VANETs: Vehicular Ad-hoc Networks
MANETs: Mobile Ad-hoc Networks
WAT: Wireless Access Technology
POC: Point of Contact
CC: Central Control
NFL: No Free Lunch
PDF: Probability Density Function
